# The Role of the Glutamate–Glutamine Cycle in Synaptic Transmission During Ischemia and Recovery

**DOI:** 10.1111/ejn.70604

**Published:** 2026-07-10

**Authors:** Hannah van Susteren, Christine R. Rose, Michel J. A. M. van Putten, Hil G. E. Meijer

**Affiliations:** ^1^ Department of Applied Mathematics University of Twente Enschede the Netherlands; ^2^ Institute of Neurobiology Heinrich Heine University Düsseldorf Germany; ^3^ Clinical Neurophysiology Group, Department of Science and Technology University of Twente Enschede the Netherlands; ^4^ Medisch Spectrum Twente Enschede the Netherlands

**Keywords:** cerebral ischemia, computational model, glutamate, glutamine, neurotransmitter, synaptic transmission

## Abstract

Cerebral ischemia impairs neuronal and glial function, ranging from transient synaptic failure to irreversible damage. The effects of ischemia on excitatory synaptic transmission remain incompletely understood. Here, we present a detailed biophysical model, including the first full implementation of the glutamate–glutamine cycle (GG‐cycle), which is essential for proper functioning of glutamatergic synapses. We model a presynaptic neuron and an astrocyte in a finite extracellular space (ECS), surrounded by an oxygen bath as a proxy for energy supply. The model includes ionic currents with corresponding channels and transporters such as the sodium‐potassium ATPase. To model synaptic transmission, we combine calcium‐dependent glutamate release, its uptake by the sodium‐dependent excitatory amino acid transporters (EAATs), and the GG‐cycle, including glutamine synthesis. We simulate ischemia by blocking energy supply completely. This drives the neuron into depolarization block, with pathological ion concentrations and extracellular glutamate accumulation despite disrupted synaptic release. Synaptic transmission failure is not primarily caused by excessive glutamate release or by failure of glutamine synthetase, but mainly results from EAAT dysfunction, driven by the collapse of the sodium gradient. Restoring synaptic transmission is not possible by solely targeting glutamate dynamics but is possible by restoring ion gradients by inhibition of the voltage‐gated Na^+^‐channel. Our study highlights the critical role of ion homeostasis, in particular the sodium gradient, in failure and recovery of synaptic function and the EAAT during metabolic stress.

## Graphical abstract

We show that severe ischemia results in loss of ion homeostasis, extracellular glutamate accumulation and disrupted glutamate release. Synaptic transmission failure is mainly caused by reduced astrocytic EAAT‐mediated glutamate uptake, driven by the collapse of the sodium gradient. Modulation of glutamate dynamics alone is insufficient for recovery; restoration of ionic gradients is essential for the recovery of synaptic transmission.

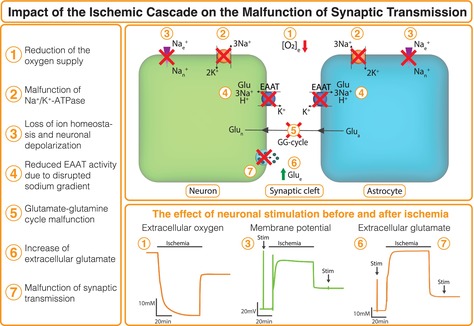

**Abbreviations**
EAAT: excitatory amino acid transporter
ECS: extracellular space
GG‐cycle: glutamate–glutamine cycle
GS: glutamine synthetase
NKA: Na^+^/K^+^‐ATPase

## 1 Introduction

Synaptic transmission is essential for normal brain function and critically depends on sufficient energy supply. During cerebral ischemia, synaptic transmission is one of the first processes to fail (Hofmeijer and van Putten 2012), affecting both excitatory and inhibitory neurotransmission. Disruption of synaptic transmission may lead to excitotoxicity and ultimately neuronal injury or death (Choi 1988; Olloquequi et al. 2018; Martinez Cruz et al. 2017). Impaired synaptic transmission includes dysregulation of glutamate homeostasis, characterized by accumulation of extracellular glutamate and depletion of glutamine (Choi 1988; Benveniste et al. 1984). Furthermore, disrupted glutamate homeostasis is closely associated with dysfunction of the glutamate–glutamine cycle (GG‐cycle), a relatively understudied, energy‐dependent process that couples astrocytic glutamate uptake, glutamine synthesis, and neuronal glutamate recycling (Bak et al. 2006; Tani et al. 2014).

Under physiological conditions, excess extracellular glutamate is primarily cleared by the astrocytic excitatory amino acid transporters (EAATs) (Mahmoud et al. 2019). The main pathway for astrocytic glutamate is the GG‐cycle (Bak et al. 2006; Lemberg and Alejandra Fernández 2009; Waagepetersen et al. 2005) in which astrocytic glutamate is converted into glutamine by the energy‐dependent enzyme glutamine synthetase (GS) (Lieth et al. 2001; Petito et al. 1992; Bak et al. 2006). Following synthesis, glutamine is transported out of astrocytes and taken up by neurons (Bröer and Brookes 2001; Chaudhry et al. 2002; Yao et al. 2000; Mackenzie et al. 2003; Todd et al. 2017), where it is converted back into glutamate (Kvamme et al. 2000; Kvamme et al. 2001). In the neuron, vesicle recycling replenishes the neuronal depot, enabling glutamate release during neuronal activity. While the essential role of the GG‐cycle in regulating synaptic transmission under physiological conditions is well understood, its role during ischemia and subsequent recovery remains unclear.

Biophysical models are powerful tools for exploring the complex interactions underlying synaptic transmission failure during ischemia, as they enable simultaneous analysis of neurotransmitter dynamics and associated ion fluxes. Furthermore, computational models readily allow simulation of perturbations in a single component, such as a transporter or enzyme, to study its effect on the whole system. We build on previous computational studies of single synapses focusing on glutamate dynamics. For instance, studies on single synapses have demonstrated the importance of astrocytic glutamate uptake and its impact on postsynaptic receptors (Allam et al. 2012; Dronne et al. 2007). Other studies have explored the turnover rates of glutamate and glutamine in isolated systems (Shen 2013). Subsequent research has extended these models to include ion dynamics in the tripartite synapse, e.g., highlighting the critical consequences of increased astrocytic sodium concentrations due to increased EAAT activity, which can reverse the action of the sodium‐calcium exchanger (NCX) (Breslin et al. 2018). Additionally, impaired glutamate uptake has been shown to prolong the duration of spreading depression and prevent recovery (Hübel et al. 2017), whereas elevated astrocytic glutamate concentrations alter the temporal profile of glutamate in the extracellular space (ECS) (Flanagan et al. 2018). Finally, detailed modeling of the tripartite synapse, including ion and glutamate dynamics during ischemia, identified key factors for reversible or irreversible synaptic damage (Kalia et al. 2021).

Although these studies provide valuable insights into synaptic transmission, none have incorporated the full dynamics of the GG‐cycle alongside ion dynamics. To better understand synaptic failure during metabolic stress, we present a detailed biophysical model that integrates the GG‐cycle with ionic fluxes across three synaptic compartments. To our knowledge, this is the first comprehensive simulation of both glutamate dynamics and ion fluxes in this setting.

Our model comprises a neuron, an astrocyte, and a finite ECS. Ion movement is based on electrodiffusion. Synaptic transmission includes calcium‐dependent vesicle recycling and a novel implementation of the GG‐cycle. Cell swelling is modeled using passive water influx driven by osmotic pressure gradients. Finally, to simulate varying degrees of energy deprivation, we incorporate oxygen dynamics along with an external oxygen bath that allows diffusion into the ECS. With this model, we examine the regulation of synaptic transmission by the GG‐cycle during ischemia and recovery. The model predicts that glutamate accumulation during severe ischemia primarily results from impaired astrocytic EAAT‐mediated uptake, caused by disrupted ion gradients. Recovery of synaptic transmission requires restoration of ion gradients rather than direct intervention in glutamate dynamics.

## 2 Methods

**FIGURE 1:**
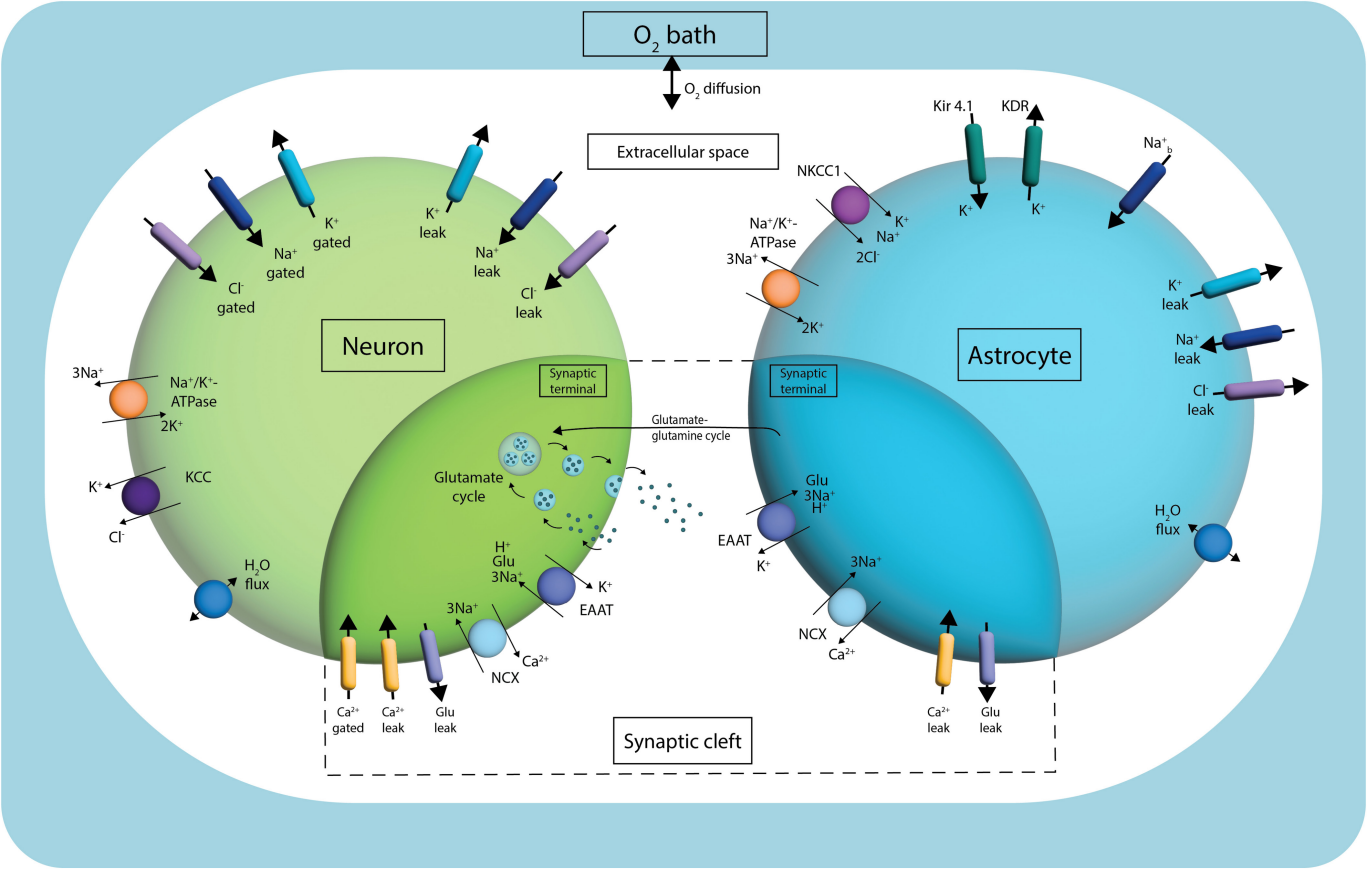
Schematic overview of the model. The model includes six compartments: three main compartments for the neuron, astrocyte, and ECS; and three synaptic compartments: the neuronal and astrocytic synaptic terminal and the synaptic cleft. In the main compartments, sodium, potassium, and chloride dynamics are considered. In the synaptic compartments, calcium, glutamate, and glutamine concentrations are additionally included. Glutamate and glutamine dynamics in the astrocyte and neuron are described by the GG‐cycle. The ECS is surrounded by an oxygen bath that allows oxygen diffusion between the bath and ECS. The main ion transporters are the energy‐dependent Na^+^/K^+^‐ATPase (NKA) and the Na^+^‐dependent glutamate transporter excitatory amino acid transporter (EAAT), located in the main compartment and synaptic compartment, respectively. KCC: K^+^/Cl^−^‐cotransporter, NKCC1: Na^+^/K^+^/Cl^−^‐cotransporter, Kir4.1: K^+^ inward rectifier channel 4.1, NCX: Na^+^/Ca^2+^‐cotransporter. KDR: K^+^ delayed rectifying current.

**FIGURE 2:**
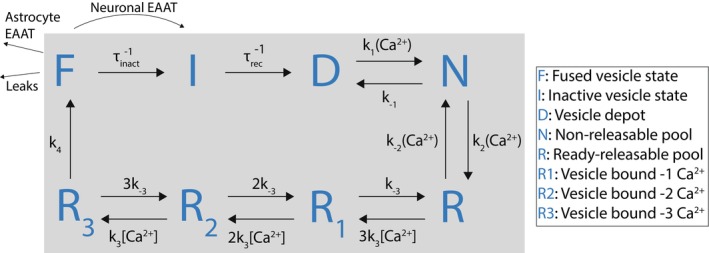
Overview of the glutamate cycle from Kalia et al. (2021). The cycle consists of eight states with reaction rates $\tau_{rec},k_{i}$, which are forward or backward rates and can be calcium‐dependent. Starting at the glutamate depot, where most neuronal glutamate is located, glutamate moves through five calcium‐dependent states ($N,R,R_{1},R_{2}$ and $R_{3}$) before it is released into the ECS. From the fused state F, which represents extracellular glutamate, the EAAT transports glutamate into the first state in the neuron, the inactive vesicle state. From the inactive state, glutamate is packed into vesicles and transported to the depot.

**FIGURE 3:**
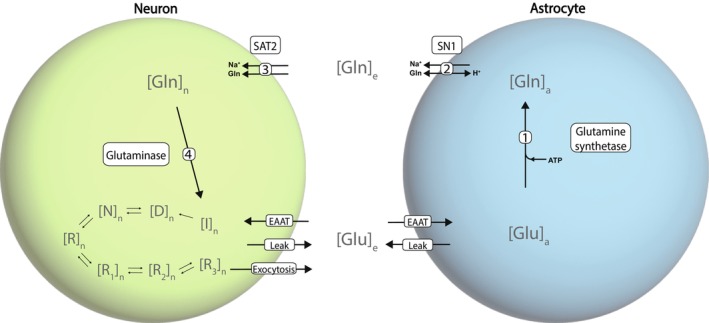
Overview of the glutamate–glutamine cycle and the glutamate cycle. The glutamate–glutamine cycle consists of four steps. First, astrocytic glutamate is converted into glutamine by the enzyme glutamine synthetase, an ATP‐dependent step. Second, astrocytic glutamine along with sodium is transported to the extracellular space by the system N transporter (SN1). Third, from the extracellular space, sodium and glutamine are transported into the neuron by the system A transporter (SAT2). Lastly, neuronal glutamine is converted back into glutamate by the enzyme glutaminase and transported to the glutamate cycle, specifically the inactive state. From here, glutamate can move through all states of the glutamate cycle before it is excreted to the extracellular space.

We elaborate on the model of Kalia et al. (2021) and consider a glutamatergic presynaptic neuron and an associated astrocyte confined in a finite ECS, shown in Figure 1. The ECS is surrounded by an oxygen bath that allows oxygen diffusion to the ECS. In this model, oxygen serves as a proxy for ATP and is considered to be the energy source. Transmembrane ion fluxes for sodium, potassium, and chloride are described by voltage‐gated channels and leak channels. To maintain ion homeostasis, the transmembrane ion fluxes are counteracted by ion transporters, such as the energy‐dependent sodium‐potassium ATPase (NKA), the potassium‐chloride transporter (KCC), and the sodium‐potassium‐chloride cotransporter (NKCC1). Volume regulation is based on differences in osmotic pressure, which can lead to cell swelling. Calcium dynamics in the synaptic compartments are described by voltage‐gated and leak channels, supported by the NCX. To simulate stimulation experiments, a square‐wave sodium current is applied, used for action potential generation. To model synaptic transmission, we introduce the first complete overview of glutamate–glutamine dynamics. For glutamate endocytosis and exocytosis, we combine calcium‐dependent glutamate release (Figure 2) with uptake by the EAAT. To complete glutamate recycling, we implement the GG‐cycle, depicted in Figure 3. In this cycle, astrocytic glutamate is converted into glutamine by the ATP‐dependent enzyme GS (Schousboe et al. 2014; Petito et al. 1992). Subsequently, glutamine is transported via ECS back to the neuron by the SN and SAT transporters (Bröer et al. 2002; Bröer and Brookes 2001; Chaudhry et al. 2002; Todd et al. 2017; Yao et al. 2000; Solbu et al. 2010), also known as SNAT3 and SNAT2, respectively. Finally, glutamine is converted back into glutamate by the enzyme glutaminase and glutamate moves through the calcium‐dependent glutamate cycle (Kalia et al. 2021; Bak et al. 2006; Krebs 1935), where it is stored in the neuronal depot. Ischemia is simulated by lowering the oxygen concentration in the bath manually. Due to unrestricted exchange between the bath and the ECS, this results in restricted energy supply for the neuron and astrocyte. Different severities of ischemia are simulated by changing the duration of reduced oxygen and thereby the level of oxygen in the bath.

### 2.1 The Model Equations

The model equations are based on the rate of change of the molar amount for each ion, which is determined by the sum of all currents. The gating variables for the voltage‐gated sodium, potassium, and chloride channels are based on the Hodgkin–Huxley formalism. Lastly, volume dynamics depend on the difference in osmolarity, i.e., the difference in total concentrations in each compartment. The fundamental model equations are:

(1a) $$\frac{d}{dt}N_{X}^{i}=-\frac{1}{z_{X}F}\underset{j}{\sum }I_{j}^{X,i},$$

(1b) $$\frac{d}{dt}\;q=\alpha_{q}\;(1-q)+\beta_{q}\;q,\;\text{for}\;q\in \{m,n,h\},$$

(1c) $$\frac{d}{dt}W_{i}=L_{H_{2}O}\;R\;T\underset{X}{\sum }({\left[X\right]}_{i}-{\left[X\right]}_{e}),$$

where $z_{X}$ is the valency of ion X,F is Faraday's constant, $L_{H_{2}O}$ is the neuronal membrane water permeability, and R is the universal gas constant. The number of moles for ion X in compartment i is denoted by $N_{X}^{i}$. The volume of compartment i is $W_{i}$. Subsequently, the concentration of ion X in compartment i is computed as ${\left[X\right]}_{i}=N_{X}^{i}/W_{i}$. The rate of change of the number of moles is the sum of all currents $I_{j}^{X,i}$ affecting ion species X, where j denotes the type of current. These fundamental equations are extended with the glutamate cycle and the GG‐cycle explained in further sections.

The model adheres to three conservation laws: the conservation of charge, volume, and mass, which are formulated as follows:

(2a) $$\sum_{X,i}z_{X}N_{X}^{i}=0,$$

(2b) $$\underset{i}{\sum }W_{i}=\text{constant},$$

(2c) $$\underset{i}{\sum }N_{X}^{i}=\text{constant},$$

where the conservation of charge and mass are formulated for each ion X. The conservation laws are used to compute the extracellular volume and molar amounts as follows

(3a) $$W_{e}=W_{tot}-W_{a}-W_{n},$$

(3b) $$N_{X}^{e}=N_{X}^{tot}-\underset{i\neq e}{\sum }N_{X}^{i}.$$

The membrane potentials are computed as follows:

(4) $$V_{i}=\frac{F}{C}\underset{X}{\sum }z_{X}\;N_{X}^{i}.$$

The neuron is stimulated using a sodium square‐wave pulse current $I_{stim}$, which is added to $\frac{d}{dt}N_{{\text{Na}}^{+}}^{n}$.

A complete overview of all model components is given in Supporting Information sections A and D, along with an overview of variables (Table S3), currents (Table S4), parameters (Table S5), permeabilities (Table S6), transporter parameters (Table S7), and glutamate–glutamine parameters (Table S8).

### 2.2 Oxygen Dynamics

We have adapted the oxygen dynamics from Wei et al. (2014). The ECS is enclosed by a bath with a constant oxygen concentration, enabling oxygen diffusion to the ECS. The energy‐dependent processes in the model are the neuronal and astrocytic Na^+^/K^+^‐ATPase and conversion of astrocytic glutamate into glutamine by the enzyme GS. The model is expanded with the following model equation,

(5) $$\begin{array}{cc} \frac{d}{dt}N_{O_{2}}^{e} & =-\frac{\alpha_{O_{2}}}{F}\left(I_{\text{NKA}}^{n}+I_{\text{NKA}}^{a}\right)\\ & \;-J_{GS}+\frac{1}{2}\varepsilon_{O_{2}}W_{e}\log(1+e^{2({\left[O2\right]}_{bath}-{\left[O2\right]}_{e})}). \end{array}$$

### 2.3 The Glutamate Cycle

The glutamate cycle concerns the presynaptic vesicle formation, docking, and exocytosis. Upon arrival of an action potential in the presynaptic neuron, the voltage‐gated calcium channels open, leading to a large influx of calcium. The influx of calcium phosphorylates the protein synapsin I, responsible for immobilizing vesicles (Nichols et al. 1992). Phosphorylated synapsin releases vesicles from the depot, enabling vesicles to move towards the membrane, where the SNARE protein is located. Upon calcium binding to synaptotagmin, the SNARE protein is activated and vesicles can fuse with the membrane, resulting in exocytosis of glutamate (Nichols et al. 1992). After exocytosis, approximately 10% of glutamate is taken up by EAATs into the neuron, where it is packed into vesicles by vesicular glutamate transporters (VGLUTs) and will be stored in the vesicle depot. Furthermore, a small gradient‐based leak channel is present, which resembles passive glutamate transport by, e.g., cystine‐glutamate antiporter or volume‐regulated anion channels. This cycle is captured in eight states as proposed by Kalia et al. (2021) who combined the work in (Walter et al. 2013; Tsodyks and Markram 1997). The overview of all states with corresponding kinetic rates is shown in Figure 2. The model equations for the glutamate cycle can be found in the Supporting Information sections A and D.

### 2.4 The GG‐Cycle

The other 90% of glutamate is taken up by the astrocytic EAAT and passes through the GG‐cycle, which is essential for glutamate homeostasis (Berl and Clarke 1983; Bak et al. 2006). We consider four steps in the GG‐cycle, which are shown in Figure 3. First, astrocytic glutamate is converted into glutamine by the enzyme GS. Glutamate synthesis is ATP‐dependent and will not function properly during metabolic stress (Passlick et al. 2021). After the synthesis of glutamate, glutamine is transported to the neuron via a two‐step process by system A and system N transporters. We consider the transporters SN1 and SAT2, also known as SNAT3 and SNAT2, respectively, and reviewed in (Mackenzie and Erickson 2004). The electroneutral SN1 transporter controls glutamine efflux from the astrocyte and transports sodium alongside glutamine out of the astrocyte in exchange for a proton (Chaudhry et al. 2002). This transporter is known to be pH sensitive and has been demonstrated to reverse direction at low pH levels (Chaudhry et al. 2002; Chaudhry et al. 1999). Subsequently, the SAT2 transporter takes up glutamate alongside sodium from the ECS into the neuron (Chaudhry et al. 2002). Finally, neuronal glutamine is converted to glutamate by the enzyme glutaminase (Bak et al. 2006), first reported by Krebs (1935). In conclusion, the GG‐cycle consists of four steps, including energy‐dependent glutamine synthesis.

#### 2.4.1 The GG‐Cycle Model Equations

The dynamics of all four steps are described using Michaelis–Menten (MM) kinetics. The MM‐constants for each step are extracted from experimental studies (Uwechue et al. 2012; Mackenzie et al. 2003; Yao et al. 2000; Bak et al. 2006; Todd et al. 2017; Schousboe et al. 2014; Kvamme et al. 2001). We note that in the first step, the enzyme GS requires ammonium. Ammonium is a byproduct of the glutamate synthesis in the neuron (Step 4). It is suggested that an ammonium pathway exists, along which ammonium produced in the neuron is transported back to the astrocyte where it is used for glutamine synthesis (Waagepetersen et al. 2000; Bak et al. 2006; Lieth et al. 2001). Therefore, we assume a constant ammonium concentration that is sufficient for optimal function of GS. Similarly, we assume a constant pH and thus a constant proton concentration in the astrocyte and the ECS. The full model equations of the GG‐cycle are given by,

(6a) $$J_{GS}=\varphi_{GS}\left(\frac{{\left[\text{Glu}\right]}_{a}}{{\left[\text{Glu}\right]}_{a}+K_{\text{Glu}}^{GS}}\cdot \frac{{\left[{\text{NH}}_{4}^{+}\right]}_{a}}{{\left[{\text{NH}}_{4}^{+}\right]}_{a}+K_{{\text{NH}}_{4}^{+}}^{GS}}\cdot \frac{{\left[O_{2}\right]}_{e}}{{\left[O_{2}\right]}_{e}+K_{\text{ATP}}^{GS}}\right),$$

(6b) $$J_{SN}=\varphi_{SN}\left(\frac{{\left[\text{Gln}\right]}_{a}}{{\left[\text{Gln}\right]}_{a}+K_{\text{Gln}}^{SN}}\cdot \frac{{\left[{\text{Na}}^{+}\right]}_{a}}{{\left[{\text{Na}}^{+}\right]}_{a}+K_{{\text{Na}}^{+}}^{SN}}\cdot \frac{{\left[H^{+}\right]}_{e}}{{\left[H^{+}\right]}_{e}+K_{H^{+}}^{SN}}\right),$$

(6c) $$J_{SAT}=\varphi_{SAT}\left(\frac{{\left[\text{Gln}\right]}_{e}}{{\left[\text{Gln}\right]}_{e}+K_{\text{Gln}}^{SAT}}\cdot \frac{{\left[{\text{Na}}^{+}\right]}_{e}}{{\left[{\text{Na}}^{+}\right]}_{e}+K_{{\text{Na}}^{+}}^{SAT}}\right),$$

(6d) $$J_{GM}=\varphi_{GM}\left(\frac{{\left[\text{Gln}\right]}_{n}}{{\left[\text{Gln}\right]}_{n}+K_{\text{Gln}}^{GM}}\right),$$

(6e) $$\frac{d}{dt}N_{\text{Gln}}^{a}=-A_{PreSyn}J_{SN}+J_{GS},$$

(6f) $$\frac{d}{dt}N_{\text{Gln}}^{n}=V_{PreSyn}\cdot J_{SAT}-J_{GM},$$

(6g) $$\frac{d}{dt}N_{I}=\frac{-N_{I}}{t_{rec}}+\frac{1}{F}(I_{Glu}^{l,n}-I_{EAAT}^{n})+J_{GM},$$

(6h) $$\frac{d}{dt}N_{\text{Glu}}^{e}=k_{4}N_{R_{3}}-\frac{1}{F}(I_{Glu}^{l,n}-I_{EAAT}^{n})-\frac{1}{F}(I_{Glu}^{l,a}-I_{EAAT}^{a})$$

where we assume ${\left[{\text{NH}}_{4}^{+}\right]}_{a}=0.15$ mM, and an astrocytic pH of approximately 7.2. Furthermore, $N_{I}$ refers to the molar amount of glutamate in the inactive state I, which is the first neuronal glutamate state, see Figure 2. From here, glutamate can move to the neuronal depot and subsequent glutamate states. Extracellular glutamine follows from the conservation laws.

## 3 Results

Before exploring ischemic conditions, we demonstrate the importance of the GG‐cycle for synaptic transmission by comparing physiological conditions with GS knockout conditions. This is followed by simulations of moderate and severe ischemia, demonstrating the failure of synaptic transmission and showing glutamate accumulation. Subsequently, we examine the mechanisms contributing to glutamate accumulation. Lastly, we evaluate potential strategies to restore synaptic transmission.

### 3.1 GS Knockout Compared to Physiological Conditions

**FIGURE 4:**
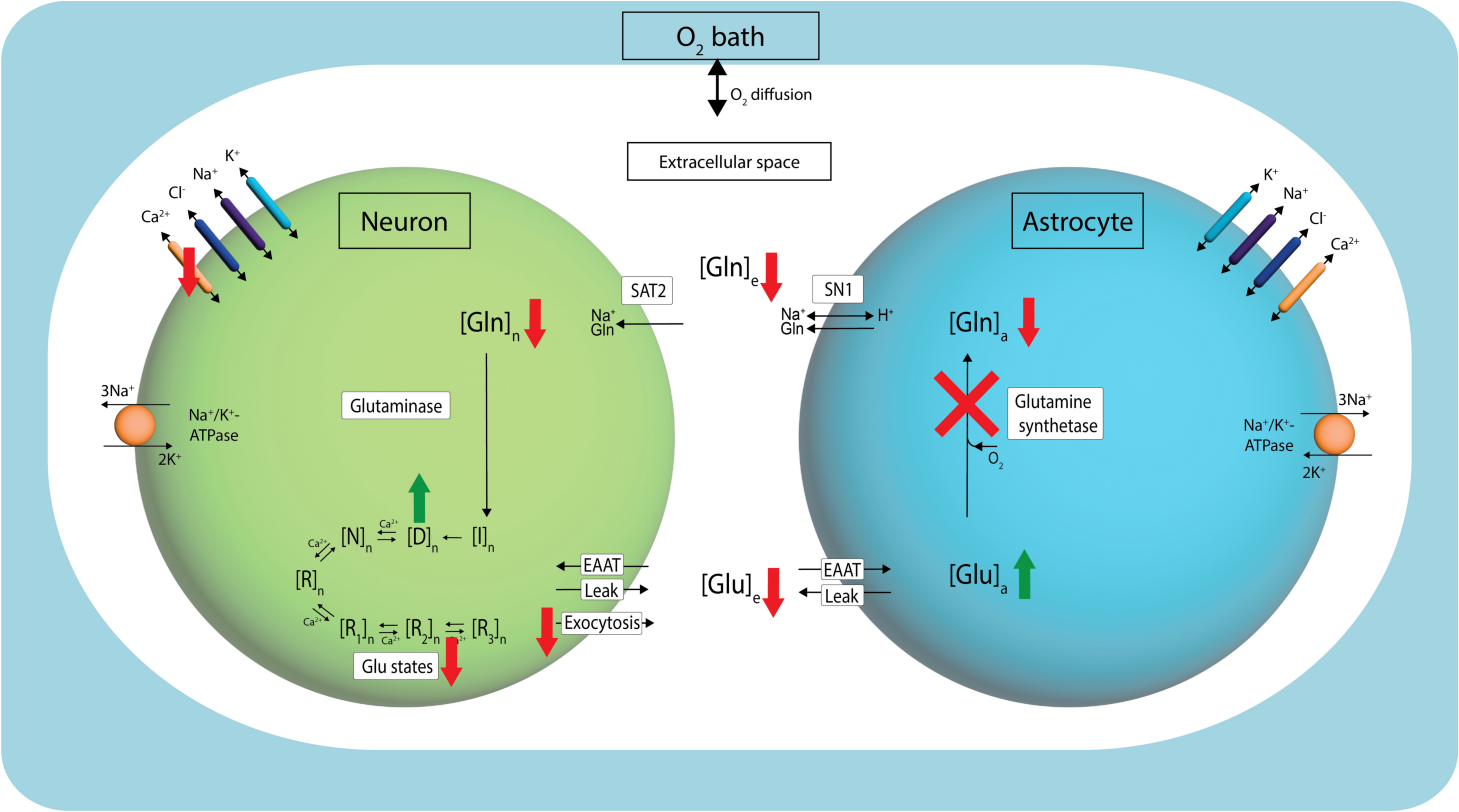
Schematic diagram of the computational model under GS knockout conditions. The glutamate–glutamine cycle is inhibited by blocking glutamine synthetase. As a result, astrocytic glutamate increases, whereas glutamine depletes and is converted into glutamate, increasing the neuronal glutamate depot. Lower neuronal calcium levels result in decreased activity of the calcium‐dependent glutamate cycle and exocytosis, leading to lower extracellular glutamate.

The computational model is summarized in Figure 4. It consists of a neuron and an astrocyte in a finite ECS. The ECS is surrounded by an oxygen bath (as a proxy for energy supply) that enables oxygen to diffuse into the ECS. Transmembrane ion transport of sodium, potassium, chloride, and calcium is regulated by voltage‐gated channels, leak channels, pumps, and ion exchangers, including the Na^+^/K^+^‐ATPase (NKA). Neuronal stimulation using an electrical current pulse to generate action potentials is modeled as a square‐wave sodium current. Synaptic transmission is modeled by combining exocytosis via calcium‐dependent vesicular glutamate release (referred to as the glutamate cycle), glutamate uptake by EAATs, and the GG‐cycle. All details of the model and its equations are given in Section 2.

**FIGURE 5:**
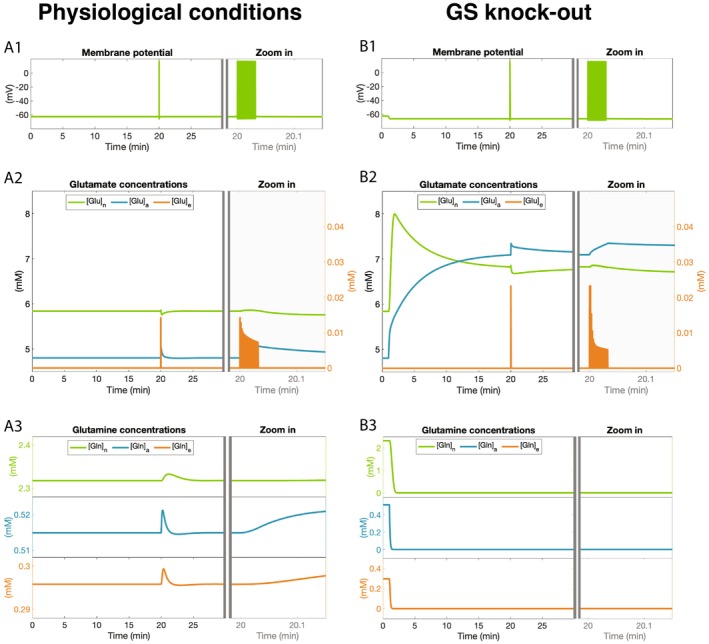
Neuronal stimulation during physiological conditions (A) and glutamine synthetase knockout conditions (B). On the left, the membrane potential (A1), glutamate concentrations (A2), and glutamine concentrations (A3) in the neuron (green), ECS (orange), and astrocyte (blue) are shown in physiological conditions. On the right, the membrane potential (B1), glutamate concentrations (B2), and glutamine concentrations (B3) in the neuron (green), ECS (orange), and astrocyte (blue) are shown in glutamine synthetase knockout conditions. External stimulation at t = 20 min during 2 s results in a burst of action potentials. Subcolumns show dynamics during the 9 s after stimulation. Blockade of glutamine synthetase at t = 1 min leads to depletion of glutamine concentrations (B3), an increased neuronal glutamate depot (B2), and increased astrocytic glutamate (B2). Generation of action potentials results in release of glutamate gradually decaying in amplitude in both physiological and knockout conditions. However, glutamate release is decreased during GS inhibition compared to physiological conditions, except during the first action potential.

The GG‐cycle is inhibited by blocking the first step of the cycle, the conversion of astrocytic glutamate into glutamine by the enzyme GS. The effect of blocking GS is illustrated in Figure 4. Neuronal stimulation in GS knockout conditions is compared to physiological conditions in Figure 5. Panel A shows the membrane potential as well as glutamate and glutamine concentrations during external electrical stimulation used to generate a burst of action potentials. In response to stimulation at t = 20 min during 2 s, a burst of action potentials is generated. The subpanels show the first few seconds after stimulation. During an action potential, calcium enters the neuron, and glutamate is released from the neuronal depot into the ECS, showing glutamate peaks, as expected (Figure 5A2). Subsequently, glutamate is taken up by the astrocytic EAAT, converted into glutamine, and transported via the GG‐cycle (Figure 5A3).

Panel B of Figure 5 shows the membrane potential and glutamate, glutamine concentrations in GS knockout conditions, simulating the effect of methionine sulfoximine (MSO) (Rothstein and Tabakoff 1984). This block results in an increase in astrocytic glutamate, as further synthesis is halted. The glutamine concentrations in all compartments deplete as there is no glutamine supply through the GG‐cycle (Figure 5B3). All glutamine is converted into glutamate by glutaminase and accumulates in the neuronal depot (Figure 5B2). As a result of ceased glutamine transporters, which transport sodium alongside glutamine, the neuronal membrane potential decreases (Figure 5B1). Stimulating the neuron under these conditions results in a burst of action potentials, but with a reduction in glutamate release. Although the neuronal glutamate depot is maintained at a high level, the activity of the calcium‐dependent glutamate cycle is lowered due to a decrease in neuronal calcium. The neuronal calcium concentration is lower due to reduced calcium inflow via the voltage‐gated calcium channel, as a result of a lower membrane potential. In summary, blockade of GS leads to malfunction of the GG‐cycle, depletion of all glutamine concentrations, and decreased glutamate exocytosis.

### 3.2 Glutamate Dynamics During Ischemia

To simulate different ischemic conditions, two variables are varied: the oxygen concentration in the bath and the duration of the ischemic insult. This results in a range of ischemic conditions that can be classified into two cases: one in which recovery after ischemia is possible, and one in which the system transitions to a pathological state from which recovery is not possible. We illustrate one example of each case.

#### 3.2.1 Moderate Ischemia

**FIGURE 6:**
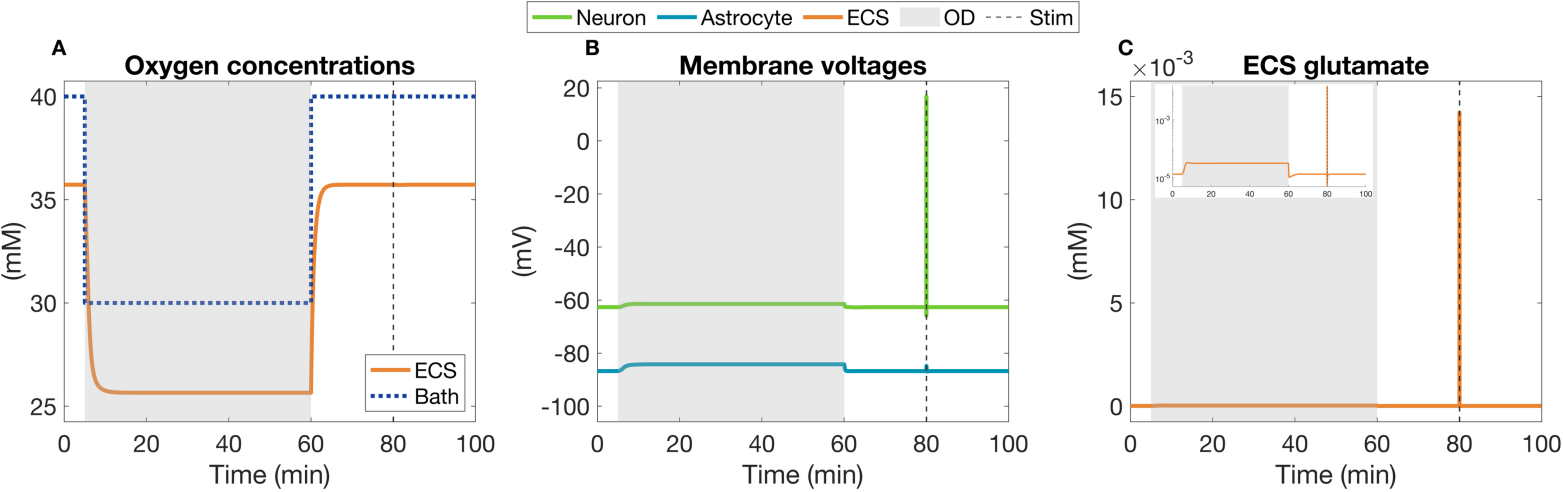
Moderate ischemia followed by brief electrical stimulation. (A) The oxygen concentration in the bath is reduced to 75% of baseline between t = 5 and t = 60 min. The extracellular oxygen concentration decreases during moderate ischemia and returns to baseline afterward. (B) Membrane potentials increase marginally. Action potentials are generated in response to an electrical current pulse at t = 80 min. (C) The extracellular glutamate concentration remains low during moderate ischemia. Glutamate is released in response to the action potential.

For moderate ischemia, we lower the oxygen concentration in the bath to 75% for 55 min, see Figure 6. During 55 min of moderate ischemia, the oxygen concentration in the ECS declines due to restricted oxygen availability from the bath and energy consumption by the neuron and astrocyte (Figure 6A). The sodium‐potassium ATPase (NKA) activity decreases, leading to temporary changes in ion concentrations. This results in a mild depolarization and a simultaneous increase in extracellular glutamate due to decreased EAAT activity. When the oxygen concentration in the bath is restored, membrane potentials (Figure 6B) and extracellular glutamate (Figure 6C) all return to their pre‐ischemia levels. To verify physiological dynamics after moderate ischemia, the neuron is stimulated with an electrical current pulse at t = 80 min for 2 s. Action potentials are generated, and the calcium‐dependent fusion of synaptic vesicles is induced, resulting in glutamate release from the neuron into the ECS. This indicates that physiological dynamics are restored, and that the system can recover from moderate ischemia.

#### 3.2.2 Severe Ischemia

**FIGURE 7:**
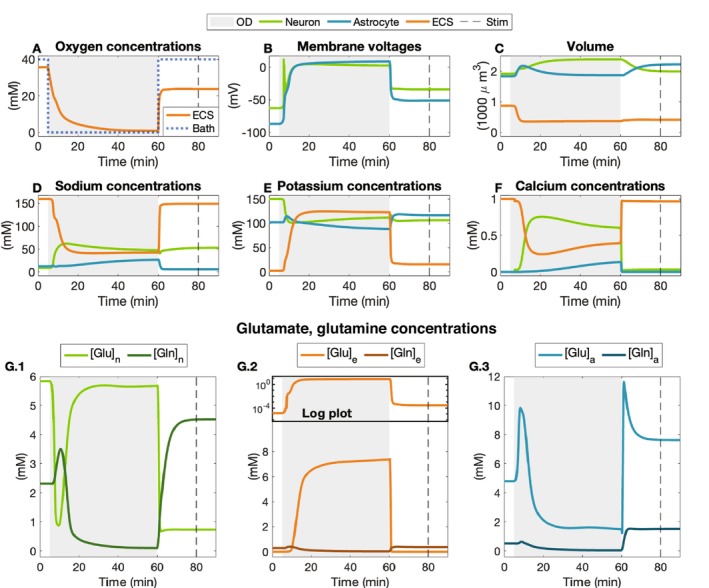
Severe ischemia followed by electrical stimulation. (A) Oxygen in the bath is completely depleted between t = 5 and t = 60 min. In response, extracellular oxygen depletes. (B) The neuron enters a depolarization block and settles in a pathological state. (C) The neuronal and astrocytic volumes increase. (D) Neuronal and astrocytic sodium increase. (E) Extracellular potassium increases significantly. (F) Neuronal and astrocytic calcium increase. (G) Dynamics of the full glutamate–glutamine cycle during ischemia, in the neuron (G.1), extracellular space (G.2), and astrocyte (G.3). Glutamate accumulates in the extracellular space in response to severe ischemia (G.2) and remains elevated after ischemia (G.2 inset). Severe ischemia results in disturbed ion homeostasis and synaptic transmission failure. Upon neuronal stimulation at t = 80 min, no action potential is generated and glutamate release is absent.

We simulate severe ischemia by reducing the oxygen level in the bath to zero for 55 min, shown in Figure 7. In response to complete energy depletion, the extracellular oxygen concentration transiently drops to zero (Figure 7A), leading to reduced activity of all energy‐dependent processes, including the neuronal and astrocytic NKA. Without functioning NKA, the neuron depolarizes, enters a period of spontaneous action potential firing, and ultimately reaches a pathological depolarized state. Concurrently, intracellular sodium and calcium and extracellular potassium increase (Figure 7D–F). These ion imbalances lead to a buildup in osmotic pressure, causing water influx and subsequent swelling of both the neuron and astrocyte (Figure 7C).

**FIGURE 8:**
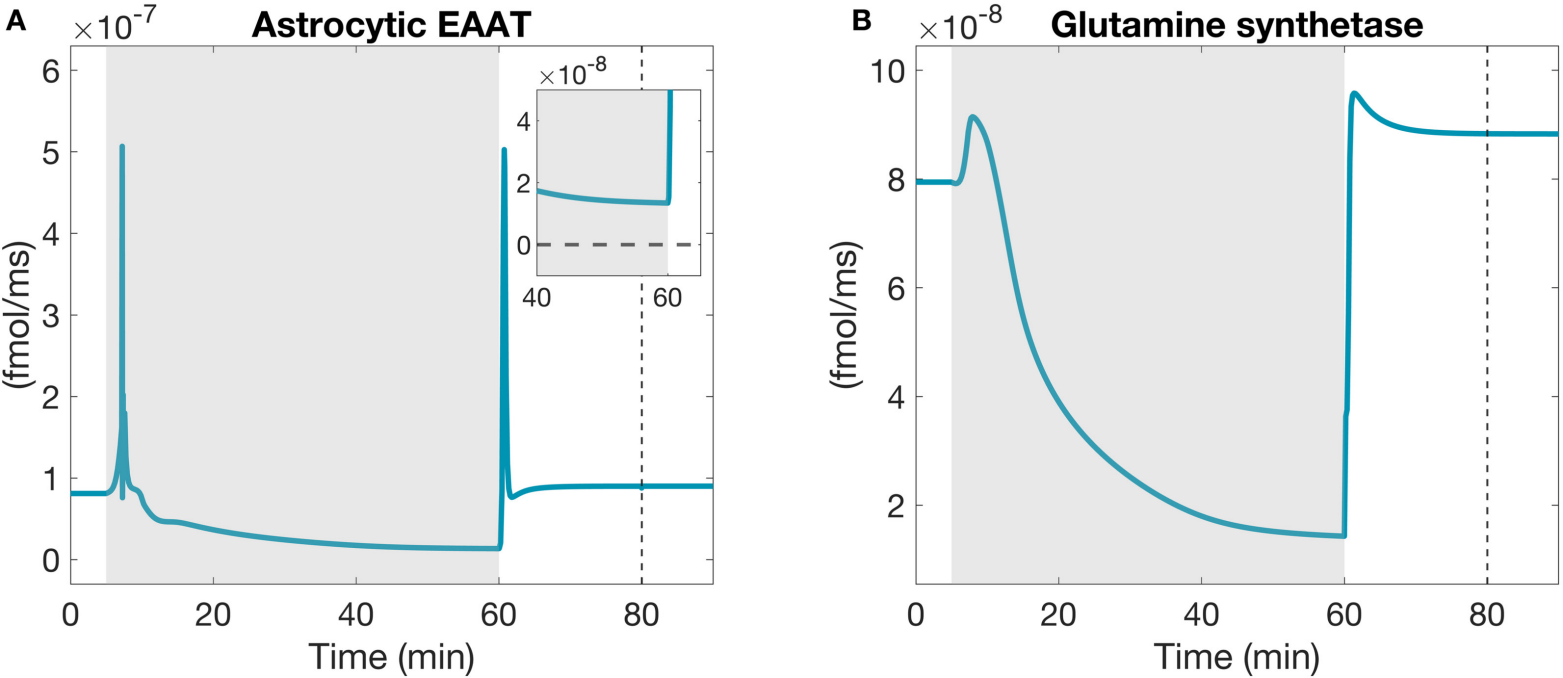
The astrocytic EAAT (A) and glutamine synthetase (B) during ischemia. Oxygen deprivation occurs between t = 5 to t = 60 min, with neuronal stimulation at t = 80 min. Failure of glutamate uptake precedes malfunction of glutamine synthesis. The astrocytic EAAT exhibits a rapid increase in activity before malfunctioning and does not reverse direction (as shown in the inset). Glutamine synthetase gradually reduces activity until malfunction.

Figure 7G shows the effects of severe ischemia on the dynamics of the GG‐cycle. In the first minutes of ischemia, glutamate is released in response to action potential generation. A fraction of extracellular glutamate is taken up by the astrocytic EAAT, which exhibits a rapid and transient peak at the start of ischemia, see Figure 8A. Initially, glutamate is still converted into glutamine in the astrocyte and transported to the ECS and neuron, showing transient increases in glutamine concentrations (Figure 7G.1–G.3). After these transients, both the EAAT and GS cease activity, see Figure 8B. As glutamate uptake failure precedes GS malfunction, this results in depletion of astrocytic glutamate and subsequently also astrocytic glutamine. Over time, all remaining glutamine is transported by the SN and SAT transporters and converted into glutamate in the neuron, until glutamine is depleted in all compartments. Due to the conversion of all glutamine into glutamate, the neuronal glutamate concentration increases. Furthermore, extracellular glutamate increases drastically up to 7.5 mM (Figure 7G.2). There are multiple processes involved in the increase of extracellular glutamate. The volume of the ECS decreases, thereby increasing extracellular concentrations. Furthermore, glutamate can leak from the neuron and astrocyte via gradient‐based leak channels. Lastly, as the neuronal calcium concentration is high during ischemia, there is a small flux of glutamate throughout the entire system. Since the EAAT activity is near zero, there is a net positive glutamate flow to the ECS during ischemia, which is not due to neuronal activity. However, the working hypothesis is that the main cause of glutamate accumulation is either a reduction of the ATP‐dependent GS, increased release of glutamate, or a reduction in its clearance. These mechanisms are examined in the following section.

Having observed the effects of severe ischemia, we study how the system reacts when we restore the oxygen supply to baseline at t = 60 minutes. In contrast to moderate ischemia, after severe ischemia, the extracellular oxygen concentration does not fully recover (Figure 7A). Although increased oxygen availability can partially enhance neuronal and astrocytic NKA activity, it is not sufficient to restore ion gradients. Both the neuron and astrocyte remain depolarized, and cell swelling persists (Figure 7B–C). Similarly, the GG‐cycle does not recover after energy supply has returned (Figure 7G). Due to partial activity of the NKA and changes in ion concentrations, glutamate uptake transiently increases, leading to an increase in astrocytic glutamate. Despite limited energy available, GS resumes activity and converts glutamate into glutamine, increasing all glutamine concentrations. The neuronal glutamate concentration remains low despite increased synthesis by glutaminase. Glutamate leaves the neuron through calcium‐dependent exocytosis, which is partly active due to slightly elevated calcium after ischemia. Furthermore, glutamate flows to the ECS via the gradient‐based leak channel. The glutamate efflux processes act on a faster timescale than the influx driven by the GG‐cycle. Consequently, all glutamine concentrations increase, but the neuronal glutamate concentration does not. Lastly, the neuronal and astrocytic EAATs are unable to clear the remaining glutamate from the ECS, resulting in a residual concentration of 0.3 μM. Overall, although the oxygen supply is restored, the neuron and astrocyte remain depolarized and are unable to return to their physiological state.

To test synaptic transmission following ischemia, the neuron is stimulated with a short external current at t = 80 min. No action potentials are generated, and glutamate release is absent. In conclusion, 55 min of severe ischemia result in complete synaptic transmission failure.

### 3.3 Glutamate Accumulation During Ischemia

Our simulations show that there is significant glutamate accumulation in the ECS during ischemia, and that synaptic transmission failure is irreversible after ischemia. We explore three candidate mechanisms that may be involved in the accumulation of glutamate: a reduction of the ATP‐dependent GS, increased release of glutamate, or a reduction in its clearance. In order to explore these mechanisms, we first design a protocol in which we make GS independent of energy to study its energy dependence. Secondly, we focus on glutamate release associated with neuronal activity. Lastly, we design a protocol in which we increase the EAAT conductance.

**FIGURE 9:**
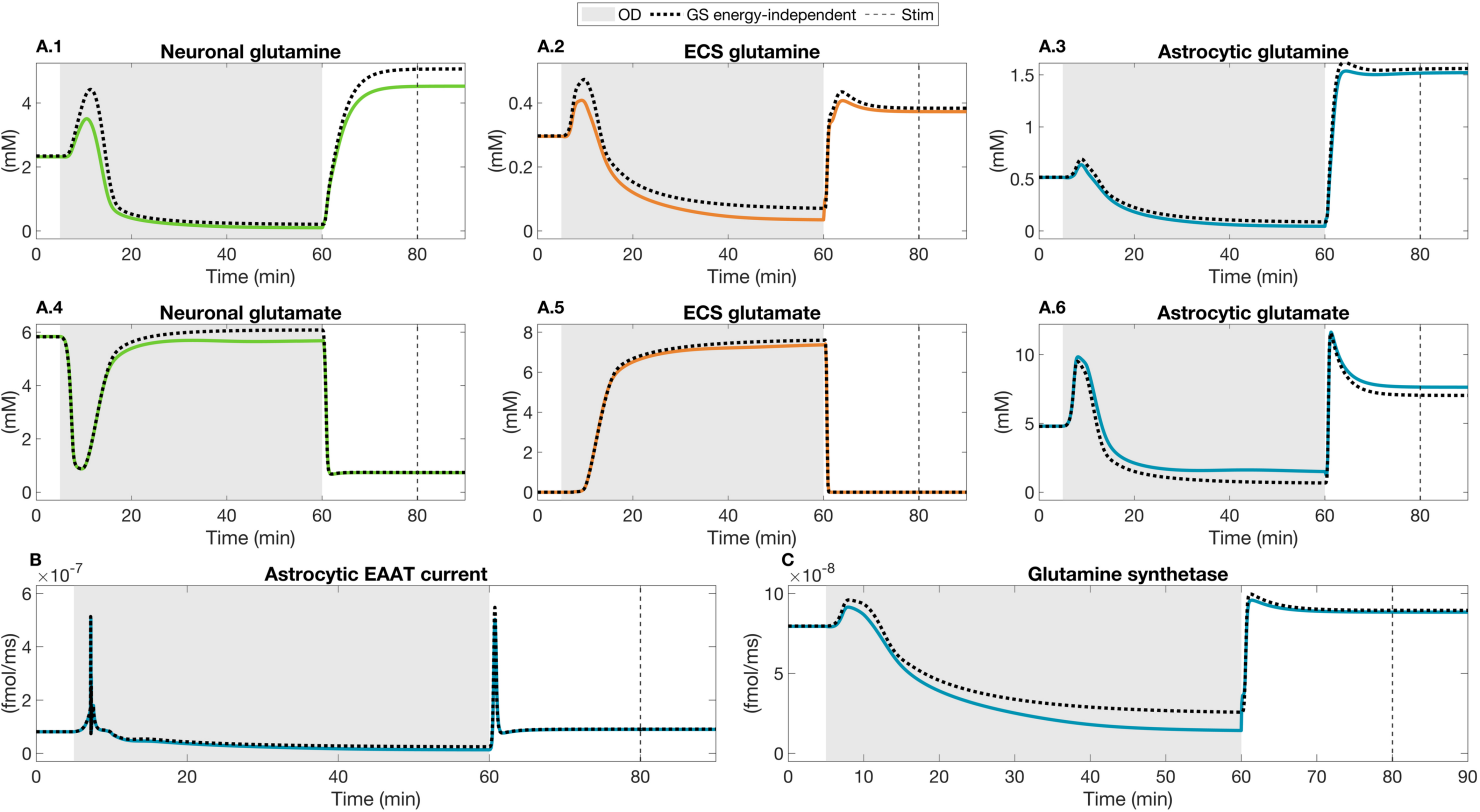
Simulation of energy‐independent glutamine synthetase. Black dashed lines indicate the new simulation of severe ischemia with an energy‐independent variant of glutamine synthetase, colored lines indicate severe ischemia in default conditions. Energy‐independent glutamine synthetase causes an increase in the extracellular glutamate accumulation. (A.1–A.6) Glutamate and glutamine dynamics. (B) The astrocytic EAAT transporter current. (C) The glutamine synthetase current.

We hypothesized that the energy dependence of GS may be a significant factor contributing to the glutamate buildup during ischemia (Lemberg and Alejandra Fernández 2009; Petito et al. 1992). To test this hypothesis, we simulate severe ischemia with an energy‐independent variant of GS, shown in Figure 9. GS remains slightly more active when it does not depend on energy (Figure 9C), resulting in a lower astrocytic glutamate concentration (Figure 9A.6). Importantly, the astrocytic EAAT current still remains impaired. Astrocytic glutamine surplus enters the GG‐cycle and is eventually converted back to glutamate, which accumulates in the ECS since glutamate uptake remains impaired (Figure 9A.5,B). Thus, an energy‐independent variant of GS aggravates glutamate accumulation in the ECS. This demonstrates that malfunction of GS is not completely due to oxygen depletion, and that the malfunction of ATP‐dependent GS is not the main cause of glutamate accumulation during ischemia.

**FIGURE 10:**
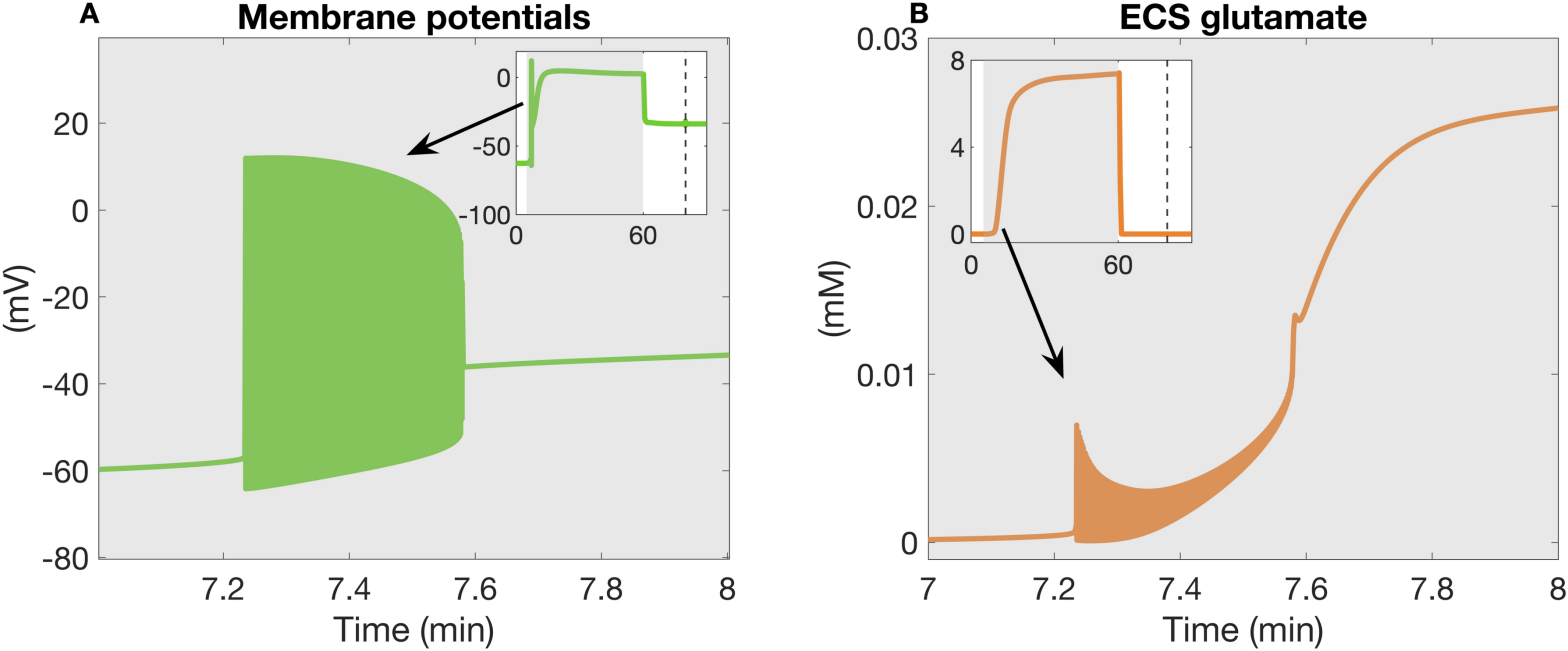
Glutamate exocytosis during ischemia. The neuronal membrane potential (A) and the extracellular glutamate concentration (B) during severe ischemia, with a detailed view of the first few minutes. The rise in extracellular glutamate during initial neuronal action potential activity is minimal.

Glutamate accumulation could result from increased glutamate release, either through neuronal activity or EAAT reversal. A burst of neuronal action potentials is observed during the first minutes of ischemia (Figure 10A). During the burst, glutamate is actively released through calcium‐dependent exocytosis. However, the extracellular glutamate concentration only increases up to 0.01 mM during the neuronal activity (Figure 10B). In the remaining period of oxygen deprivation, the glutamate concentration continues to rise to 7.5 mM, reaching extremely high levels. Moreover, no glutamate is released by the EAAT transporter since it does not reverse under ischemic conditions (Figure 8A). Thus, neither synaptic glutamate release nor EAAT reversal contributes substantially to glutamate accumulation.

**FIGURE 11:**
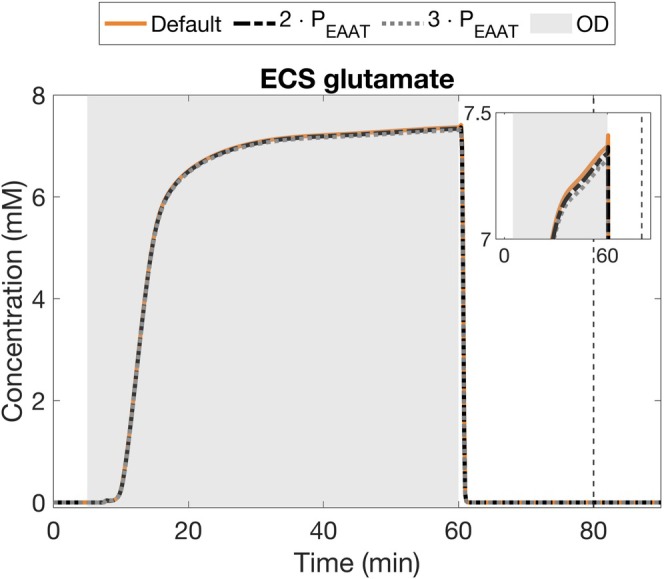
Extracellular glutamate during ischemia with increased EAAT conductance. The default simulation of ischemia (orange) is compared to simulations in which the EAAT conductance is increased twofold (black) and threefold (gray) during ischemia. Increasing the EAAT activity reduces glutamate accumulation slightly.

Since elevated extracellular glutamate levels cannot be fully explained by increased glutamate release, EAAT reversal, or impaired GS activity, we hypothesize that the primary cause is impaired glutamate uptake by the astrocytic EAAT. To verify this, we simulate severe ischemia with the EAAT conductance being doubled or tripled, shown in Figure 11. The increased EAAT activity results in only a marginal reduction of extracellular glutamate. An increase in transport conductance is insufficient to recover glutamate uptake. We infer that impairment of the EAATs plays an important role in glutamate accumulation.

**FIGURE 12:**
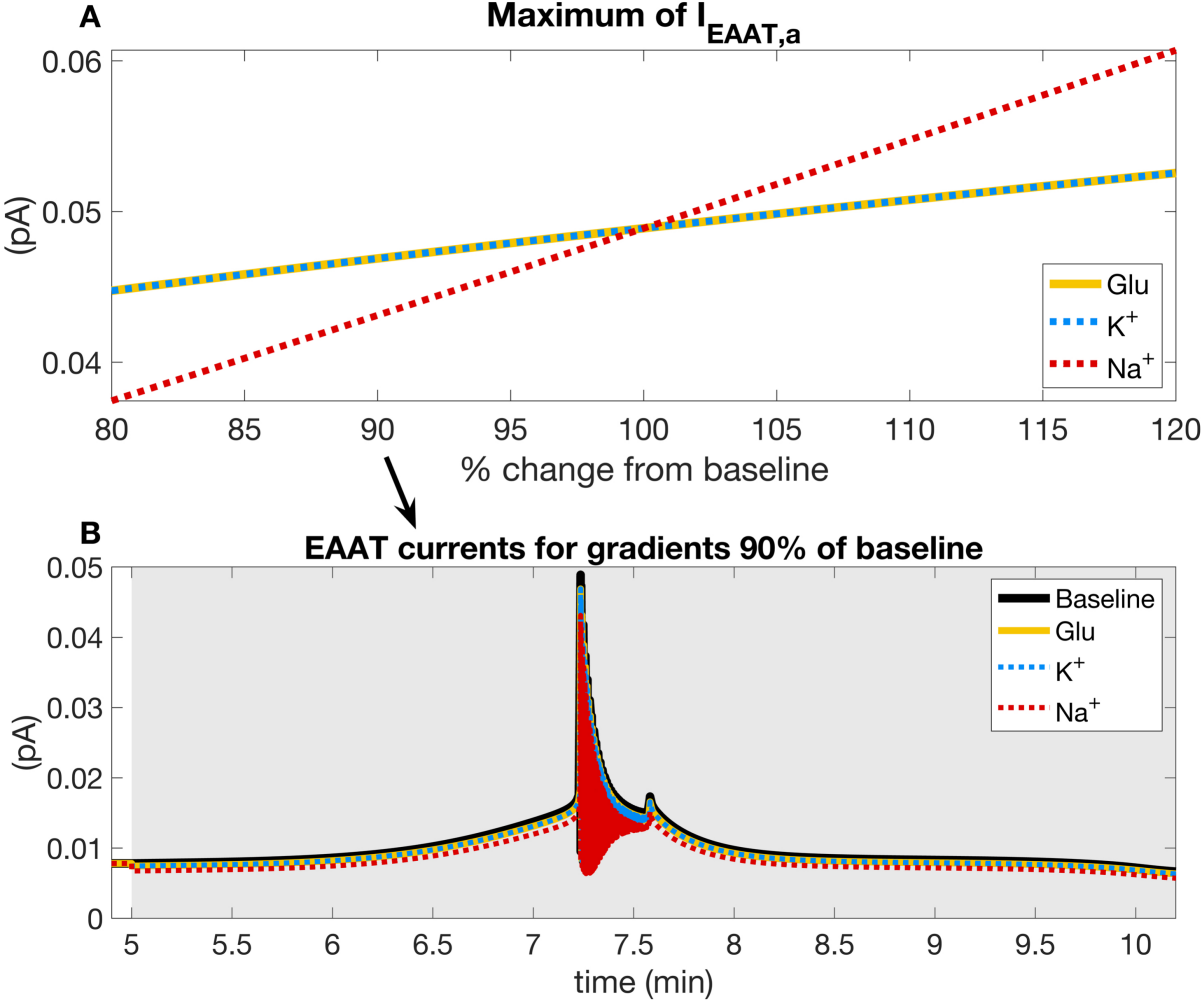
Altered EAAT currents during ischemia. (A) The maximum of the EAAT current during severe ischemia for changes in the glutamate (yellow), potassium (blue), and sodium (red) gradient compared to baseline. (B) The EAAT current for a 10% reduction in the glutamate, potassium, and sodium gradient during the first 5 min of ischemia.

To determine the main factor of insufficient clearance, we analyze the three driving forces behind the EAAT: the sodium, potassium, and glutamate gradients. We alter each gradient individually by an at most 20% increase or decrease to assess their influence on the EAAT current. To compare the effect of changes in the gradient, we measure the maximum value of the EAAT current, which is around t = 7.3 min. Figure 12A shows the maximum value for changes in the glutamate, potassium, and sodium gradient, ranging from a 20% reduction to a 20% increase. Figure 12B shows the EAAT currents in case each gradient is reduced by 10%. Modifying the potassium or glutamate gradient has minimal impact on the EAAT current, whereas altering the sodium gradient significantly affects the EAAT current, which is related to its 3:1:1 stoichiometry. These findings indicate that insufficient glutamate clearance, governed by the breakdown of the astrocytic sodium gradient, is the primary cause of glutamate accumulation.

### 3.4 Synaptic Recovery

In addition to investigating the cause of glutamate accumulation during ischemia, we also examine which mechanisms can contribute to the recovery of synaptic transmission and restoration of the GG‐cycle. We study three possible recovery mechanisms: increasing the GS activity, increasing the EAAT activity, and blocking voltage‐gated ion channels.

**FIGURE 13:**
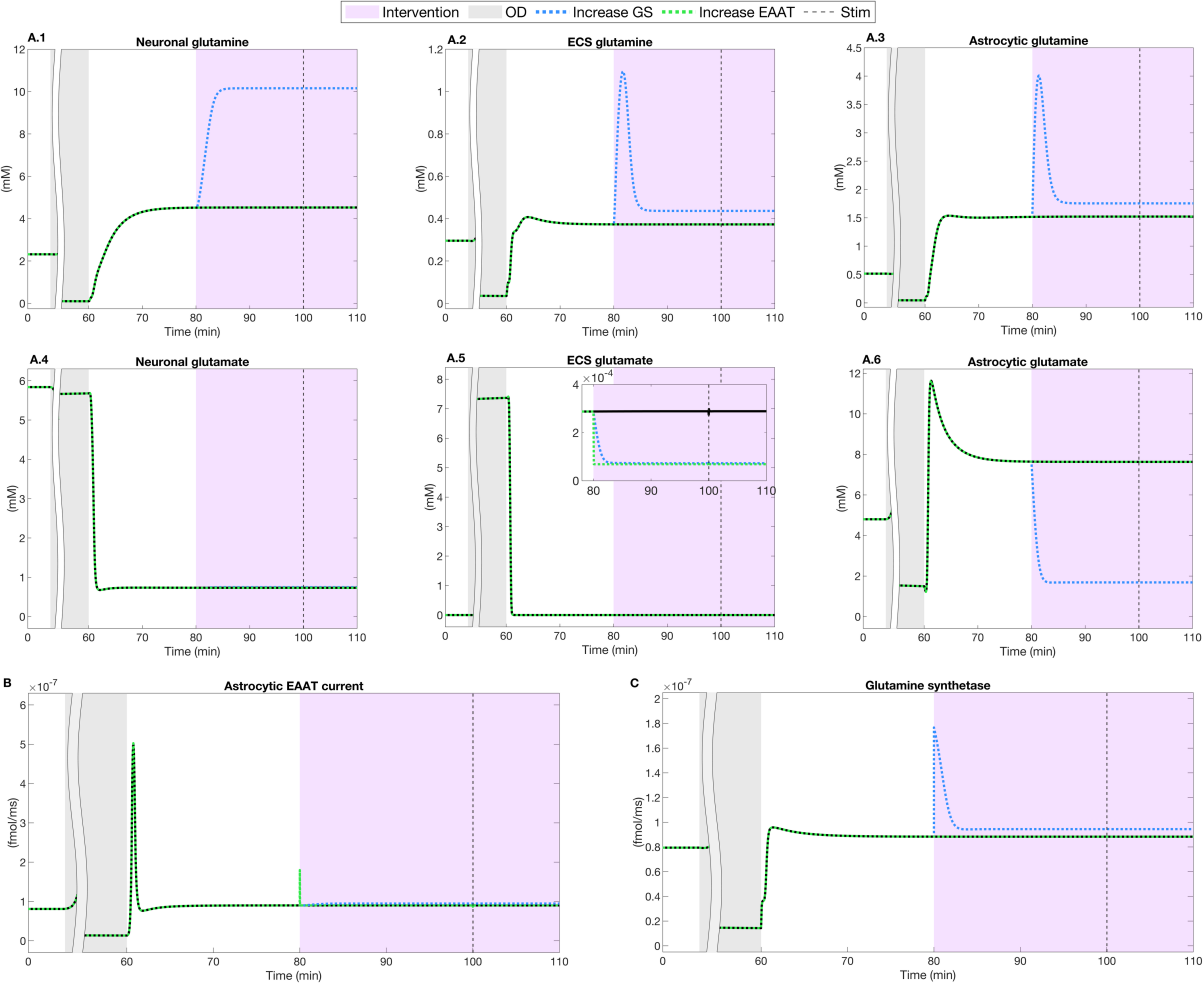
Possible recovery mechanisms after ischemia. The axis break represents the period of severe ischemia from t = 5 to t = 60 min, which is shown in Figure 7. At t = 80, the activity of glutamine synthetase (blue) and the neuronal and astrocytic EAAT currents (green) are increased. Increasing the glutamine synthetase flow rate leads to higher glutamine concentrations and reduced astrocytic and extracellular glutamate. Increasing the EAAT conductances results in a marginal increase in glutamine concentrations and reduced extracellular glutamate. At t = 100 min, the neuron is stimulated but no glutamate is released in either case. Neither of the interventions results in recovery of synaptic transmission.

In Figure 13, we show the effect of increasing the GS flow rate. The astrocytic glutamate concentration decreases, whereas all glutamine concentrations increase, but none return to their pre‐ischemic levels. Extracellular glutamate is able to decrease to 70 nM but does not fully return to its baseline concentration of 13 nM. At t = 100 min, we apply external stimulation to the neuron to test glutamate release. Although extracellular glutamate is partially cleared, synaptic glutamate release remains impaired.

Increasing the EAAT conductances has less impact on the glutamate and glutamine concentrations. The astrocytic glutamate concentration, and all glutamate concentrations increase marginally but do not return to pre‐ischemic levels, see Figure 13. Extracellular glutamate decreases, but again no glutamate is released during neuronal stimulation. Our findings indicate that even improved glutamate clearance is insufficient for recovery of synaptic transmission after ischemia.

**FIGURE 14:**
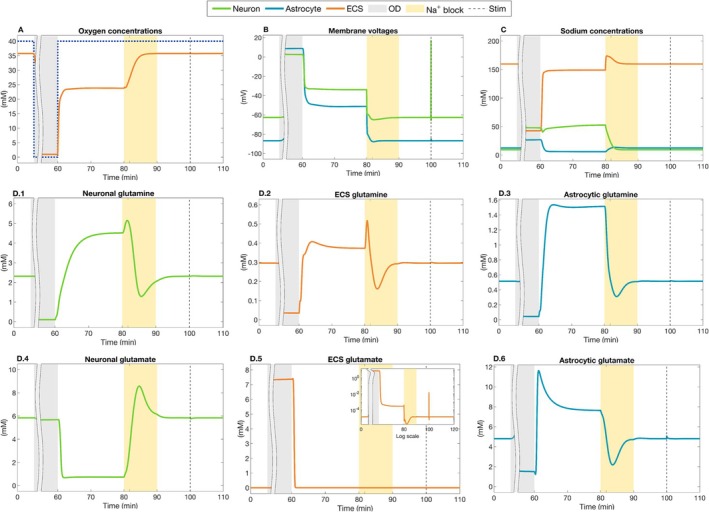
**Sodium block after ischemia.** The axis break represents the period of severe ischemia from t = 5 to t = 60 minutes, which is shown in Figure 7. From t=80 to t=90 min, voltage‐gated sodium channels are blocked. (A) The extracellular oxygen concentration returns to baseline during the blockade. (B) Membrane potentials return to the physiological equilibrium. At t=100 min, action potentials are generated in response to external stimulation. (C) Neuronal sodium can recover due to the NKA and blocked sodium influx. (D) All glutamate and glutamine concentrations return to baseline during the sodium channel block. The inset in (D.5) shows extracellular glutamate on a log‐scale. At t=100 min, the neuron is stimulated and glutamate is released into the extracellular space. Blockade of the neuronal sodium channels results in recovery of ion homeostasis and synaptic transmission.

We showed that the reduced sodium gradient is the main factor involved in the malfunction of the EAAT and synaptic transmission failure. We hypothesized that blockade of the TTX‐sensitive voltage‐gated sodium channels might rescue the neuron and astrocyte from their pathological equilibrium. The neuronal sodium channel is blocked to prevent sodium influx from t = 80 to t = 90 min, shown in Figure 14. In this scenario, the neuronal NKA restores the sodium and other ion gradients, membrane potentials return to baseline values, and extracellular oxygen concentrations are restored (Figure 14A–C). Due to the sodium dependence of glutamate uptake and glutamine transport, all glutamate and glutamine concentrations return to their pre‐ischemic values (Figure 14D). The inset in Figure 14D.5 shows extracellular glutamate on a logarithmic scale to better highlight the difference between pre‐ and postischemic concentrations. In response to an electrical current at t = 100 min, the generation of an action potential activates the calcium‐dependent glutamate cycle, and glutamate is released into the ECS. Altogether, our results show that impaired synaptic transmission during ischemia results from EAAT dysfunction, which is governed by the sodium gradient. If normoxia is restored, restoration of the sodium gradient by inhibition of the voltage‐gated sodium channel is sufficient for recovery of synaptic transmission.

## 4 Discussion

In this study, we present the first detailed model of a neuron and astrocyte incorporating the complete GG‐cycle to investigate synaptic transmission during ischemia and recovery. We show that synaptic transmission failure during severe ischemia is primarily driven by collapse of ion homeostasis, in particular the sodium gradient, rather than by excessive glutamate release or primary failure of glutamine synthesis.

### 4.1 The GG‐Cycle Is Essential for Synaptic Transmission

Our biophysical model combines ion concentrations with the complete GG‐cycle, which enables a detailed analysis of synaptic transmission during ischemia. Implementation of the GG‐cycle is essential to study synaptic transmission as it provides the main pathway for astrocytic glutamate back to the neuron (Nicoli et al. 2024; Flores‐Méndez et al. 2016). Experimental studies demonstrate that inhibiting GS reduces glutamate release and thus impairs synaptic transmission (Tani et al. 2014; Rothstein and Tabakoff 1984), as we verify in Figure 5. Therefore, incorporating the essential GG‐cycle when studying synaptic transmission during ischemia seems warranted.

### 4.2 Severe Ischemia Leads to Disturbed Ion Homeostasis and Glutamate Accumulation

While moderate ischemia induces only transient changes in ion and glutamate concentrations, severe ischemia has significant and irreversible pathological consequences. The differences in neuronal and synaptic damage between moderate and severe ischemia are also observed experimentally (Winkelheide et al. 2008; Ueda et al. 1992; Zhao et al. 1998). Severe ischemia leads to a significant disturbance in ion homeostasis, as shown in Figure 7. Strongly increased intracellular sodium due to decreased NKA activity is one of the primary consequences of severe ischemia (Gerkau et al. 2017; van Putten et al. 2021). The extracellular potassium concentration increases due to decreased NKA activity, also determined experimentally (Kléber 1984). Furthermore, the simulations show highly elevated intracellular calcium concentrations, similar to elevated calcium levels observed experimentally (Jalini et al. 2016; Silver and Erecińska 1990), which affects calcium‐dependent glutamate release. In conclusion, the dynamics of ion concentrations affecting synaptic transmission align with experimental findings.

Besides disturbed ion homeostasis, an important consequence of severe ischemia is glutamate accumulation in the ECS. Glutamate accumulation in the ECS occurs in two distinct phases, as observed experimentally (Ueda et al. 1992; Wahl et al. 1994; Ziebarth et al. 2025). At the onset of ischemia, the initial phase is marked by a moderate rise in extracellular glutamate levels due to neuronal firing. The second phase occurs during the period of neuronal depolarization and depression, in which extracellular glutamate increases substantially, up to the millimolar range. Besides glutamate accumulation, we show that no glutamate is released upon neuronal stimulation, indicating synaptic transmission failure.

### 4.3 The Main Cause of Glutamate Accumulation Is Disrupted Uptake

While the exact cause of synaptic transmission failure is not yet fully understood, experimental data suggest three plausible mechanisms: decreased activity of energy‐dependent GS, excessive glutamate release by neurons or astrocytes, and reduced glutamate uptake or reverse uptake by the EAATs.

We first tested whether impaired GS activity contributes substantially to increased extracellular glutamate concentrations. To this end, we simulated severe ischemia with an energy‐independent variant of GS. Although this increased GS activity, the surplus of glutamine was converted into glutamate, which accumulated in the ECS as uptake remained impaired. Altering GS to be independent of energy thus aggravated glutamate accumulation, suggesting that its dysfunction is unlikely to be the main cause of glutamate accumulation.

Subsequently, we demonstrated that the extracellular glutamate concentration only increases marginally due to glutamate release during repetitive neuronal firing in the first several minutes of ischemia. The majority of glutamate accumulation occurs during the remaining phase of ischemia, in which neuronal activity is suppressed.

Furthermore, we showed that glutamate accumulation is not aggravated by reversed glutamate uptake, as is proposed in (Rossi et al. 2000; Grewer et al. 2008). The reversal potential of the EAAT is substantially more positive than the membrane potentials, see Figure S16. Thus, excessive glutamate release and EAAT reversal are unlikely to be the primary causes of glutamate accumulation.

As we have eliminated glutamate release and malfunction of the GG‐cycle as possible causes, the remaining potential cause is a reduction of the astrocytic EAAT current. Our findings are consistent with experimental evidence indicating that, under ischemic conditions, glutamate uptake by the EAAT is significantly limited (Bruhn et al. 2001). Our results indicate that EAAT function is predominantly governed by the sodium gradient, with minimal contribution from the potassium and glutamate gradients, as shown experimentally (Kelly et al. 2009; Rose et al. 2018). Thus, our model not only reproduces reduced EAAT‐mediated glutamate uptake during ischemia but also identifies the collapse of the sodium gradient as the main contributor to glutamate accumulation in the ECS.

### 4.4 Recovery of Synaptic Transmission Is Possible by Restoring the Sodium Gradient

We have shown that an increase in either GS activity or EAAT activity leads to a reduction of the extracellular glutamate concentration. However, none of these interventions result in the recovery of synaptic glutamate release. We therefore conclude that the modifications to the GG‐cycle are insufficient as a recovery mechanism. Since EAATs malfunction due to an altered sodium gradient, we investigate restoring the sodium gradient as a possible recovery mechanism. Indeed, blocking voltage‐gated TTX‐sensitive neuronal sodium channels results in recovery of ion homeostasis, and in turn, recovery of synaptic transmission.

### 4.5 Limitations

Several model assumptions should be considered when interpreting our findings.

First, we consider each compartment to be uniform and do not include spatial diffusion, but we take volume changes into account. As we consider only one synapse in an ECS, there is no ion or neurotransmitter spillover to other synapses. Due to the fixed total volume of the model, there is also no ion diffusion due to (re)perfusion such as in an experimental setup. As a result, ionic changes during prolonged ischemia in the brain could be even more pronounced than those observed in experiments. However, given that this modeling assumption impacts all ionic gradients uniformly, it does not compromise our main conclusions.

Second, the SN transporter, responsible for glutamine transport at the astrocytic membrane, is not able to reverse in our model. While experimental studies show that SN reversal can occur in response to pH changes (Todd et al. 2017), our model does not include proton dynamics and therefore does not account for this reversal mechanism. The most important driving force for the SN transporter is the sodium influx by the EAAT (Todd et al. 2017), and changes in the proton gradient are unlikely to have a dominant impact. Furthermore, intracellular and extracellular compartments both acidify similarly during ischemia, largely preserving the pH gradient (von Hanwehr et al. 1986; Hagberg 1985; Everaerts et al. 2023).

Third, we consider only one calcium compartment in the neuron and astrocyte. Distinguishing between the cytosol and the endoplasmic reticulum (ER) would introduce more detailed dynamics and timescales. It has been shown that calcium dynamics operate on several timescales in the cytosolic and ER (Bazargani and Attwell 2016; Cheng et al. 2006). During ischemia, the cytosolic calcium concentration increases partly due to calcium release from intracellular stores like the ER (Bodalia et al. 2013; Paschen and Doutheil 1999). The calcium compartment in our model represents the cytosol and experiences a large increase in concentration during ischemia. While the cause of increased calcium might differ in vivo, we argue that the resulting effect of elevated calcium is similar. For future work, it would be interesting to explore whether the difference between cytosolic calcium and ER stores influences the timescale and extent of synaptic transmission failure.

Lastly, our model does not include a postsynaptic neuron. In order to fully investigate synaptic transmission and glutamate toxicity during ischemia, it is necessary to analyze postsynaptic glutamate receptors such as NMDA and AMPA receptors. These receptors are overstimulated during ischemia, thereby releasing potassium into the ECS, and they might desensitize during glutamate toxicity (Olloquequi et al. 2018; Sattler and Tymianski 2001). Our simulations show an extracellular glutamate concentration of around 7.5 mM during ischemia. Due to the lack of spatial diffusion and the postsynaptic neuron, this concentration is likely to be higher than seen in experiments. However, glutamate toxicity is already observed for much lower extracellular glutamate concentrations (Michaels and Rothman 1990), thus we expect that postsynaptic receptors would be overstimulated in our simulation.

For future modeling efforts, the model can be extended to enable more detailed analysis of ischemic dynamics by incorporating additional components, including glutamate receptors and detailed ATP and adenosine dynamics. Furthermore, we believe that due to its high level of detail, the computational model is well‐suited to study single‐cell dynamics across a range of pathological conditions, including ischemia, Parkinson's disease, and epilepsy.

In conclusion, we have constructed a detailed model that includes the first implementation of the complete GG‐cycle. With the model, we simulate different severities of ischemia to investigate the primary cause of synaptic transmission failure and glutamate accumulation. We demonstrate that during severe ischemia, characterized by a transient complete cessation of energy supply, extracellular glutamate levels rise to the millimolar range, sufficient to induce glutamate toxicity. Our findings show that the main cause of glutamate accumulation during severe ischemia is the reduced activity of the astrocytic EAAT due to the collapse of the sodium gradient. Furthermore, we demonstrate that alterations to the GG‐cycle are not sufficient to mediate recovery, whereas blocking the neuronal sodium channels restores synaptic transmission.

## Author Contributions

**Hannah van Susteren:** conceptualization, investigation, methodology, software, validation, visualization, writing – original draft preparation, writing – review and editing. **Christine R. Rose:** conceptualization, funding acquisition, writing – review and editing. **Michel J. A. M. van Putten:** conceptualization, funding acquisition, supervision, writing – original draft preparation, writing – review and editing. **Hil G. E. Meijer:** conceptualization, funding acquisition, methodology, supervision, writing – original draft preparation, writing – review and editing. All authors contributed to the article and approved the submitted version.

## Ethics Statement

The authors have nothing to report.

## Conflicts of Interest

The authors declare no conflicts of interest.

## Supporting information



SupplementaryMaterials.pdf.

## Data Availability

The code for all the simulations performed is available at https://github.com/HannahvanSusteren3/GGsyntrans.
