# Supplementary material for: The Role of the Glutamate–Glutamine Cycle in Synaptic Transmission During Ischemia and Recovery
Source: Eur J Neurosci. 2026 Jul 10;64(1):e70604. doi: 10.1111/ejn.70604 (PMC13351822; doi:10.1111/ejn.70604)
Supplement: Supplementary file 1 — SupplementaryMaterials.pdf. [file EJN-64-0-s001.pdf]

A Supplementary model components

Additional model components compared to Kalia et al., 2021, that were not yet explained in the Methods section.

The astrocytic potassium delayed rectifying channel (KDR) was added to the model to provide an additional pathway for efflux of potassium in the astrocyte. The formulation is taken from (Dronne et al., 2006) and is given by:

$$\begin{cases} \alpha_{m,KDR} &= 0.0047\frac{V_a-8}{1-\exp\left(-(V_a-8)/12\right)}, \\ \beta_{m,KDR} &= \exp\left(-(V_a+127)/30\right), \\ m_{\infty,KDR} &= \frac{\alpha_{m,KDR}}{\alpha_{m,KDR}+\beta_{m,KDR}}, \\ h_{\infty,KDR} &= 1/(1+\exp(V_a+25)/4), \\ E_K &= \frac{RT}{F}\log\left([K^+]_a/[K^+]_e\right), \\ \frac{\mathrm{d}}{\mathrm{d}t}m_{KDR} &= (m_{\infty,KDR}-m_{KDR})/\tau_{KDR}, \\ \frac{\mathrm{d}}{\mathrm{d}t}h_{KDR} &= (h_{\infty,KDR}-h_{KDR})/\tau_{KDR}, \\ I_{KDR} &= P_{KDR}/1000\ \ g_{KDR}\ \ m_{KDR}^2\ \ h_{KDR}\ \ (V_a-E_K^a). \end{cases}$$

The formulation for the EAAT transporter is taken from (Breslin et al., 2018). The EAAT transporter is based on the voltage difference between the membrane potential and the reversal potential of the transporter as follows:

$$\begin{cases} f_g([Glu]_e) &= 1/(1+\exp\left(r_g(s_g-[Glu]_e)\right)), \\ V_{EAAT}^{rev,i} &= \frac{RT}{2F}\log\left(\frac{[H^+]_e}{[H+]_i}\left(\frac{[\mathrm{Na}^+]_e}{[\mathrm{Na}^+]_i}\right)^3\frac{[K^+]_i}{[K^+]_e}\frac{[Glu]_e}{[Glu]_i}\right), \\ f_v(V_i) &= -\frac{1}{6}\alpha_{EAAT,i}\left(\exp\left(\beta_{EAAT,i}(V_i-V_{EAAT}^{rev,i})\right)-1\right), \\ I_{EAAT}^i &= P_{EAAT,i}\,f_g([Glu]_e)\,f_v(V_i)\,A_{PreSyn}, \end{cases}$$

for $i = \{\mathrm{n, a}\}$ for the neuronal and astrocytic EAAT, respectively.

As the glutamate-glutamine cycle provides an additional astrocytic sodium efflux by the SN-transporter, an additional influx was needed. We implemented a straightforward sodium current $I_T^{\mathrm{Na}^+,a}$ based on the Nernst potential, similar to that in (Breslin et al., 2018), with the following formulation:

$$I_T^{\mathrm{Na}^+,a} = P_T^{\mathrm{Na}^+,a}(V_a - E_{\mathrm{Na}^+}^n)A_a$$

A.1 Basis model components

Here we give an overview of all model components that are unmodified from Kalia et al., 2021.
Voltage-gated ion channels

$$
\begin{aligned}
I_G^{\mathrm{Na}^+,n} &= P_G^{\mathrm{Na}^+,n} m^3 h \mathrm{GHK}(V_n, [\mathrm{Na}^+]_n, [\mathrm{Na}^+]_e),\\
I_G^{\mathrm{K}^+,n} &= P_G^{\mathrm{K}^+,n} n^2 \mathrm{GHK}(V_n, [\mathrm{K}^+]_n, [\mathrm{K}^+]_e),\\
I_G^{\mathrm{Ca}^{2+},n} &= P_G^{\mathrm{Ca}^{2+},n} m^2 h \mathrm{GHK}(V_n, [\mathrm{Ca}^{2+}]_n, [\mathrm{Ca}^{2+}]_e),\\
I_G^{\mathrm{Cl}^-,n} &= \frac{P_G^{\mathrm{Cl}^-,n}}{1+\exp\left(-\frac{V_n+10}{10}\right)} \mathrm{GHK}(V_n, [\mathrm{Cl}^-]_n, [\mathrm{Cl}^-]_e),
\end{aligned}
$$

where m, h and n are Hodgkin-Huxley gating variables, and GHK the Goldman–Hodgkin–Katz formulation given by

$$
\mathrm{GHK}(V_i, [X]_i, [X]_e) = \frac{\mathrm{F}^2 V_i}{z_X^2 \mathrm{RT}} \frac{[X]_i - [X]_e \exp\left(-\frac{\mathrm{F}V_i}{z_X \mathrm{RT}}\right)}{1-\exp\left(-\frac{\mathrm{F}V_i}{z_X \mathrm{RT}}\right)}.
$$

Gating variables
The components of the gating variables as shown in 1 are given by

$$
\begin{aligned}
\alpha_m &= \frac{0.32(V_n+52)}{1-\exp\big(-(V_n+52)/4\big)}, \beta_m = \frac{0.28(V_n+25)}{\exp\big((V_n+25/5)\big)-1},\\
\alpha_h &= 0.128\exp\big(-(V_n+53)/18\big), \beta_h = \frac{4}{1+\exp\big(-(V_n+30)/5\big)},\\
\alpha_n &= \frac{0.016(V_n+35)}{1-\exp\big(-(V_n+35)/5\big)}, \beta_n = 0.25\exp\left(-\frac{V_n+50}{40}\right).
\end{aligned}
$$

Leak currents in neuron and astrocyte

$$
I_L^{X,i} = P_L^{X,i} \mathrm{GHK}(V_i, [X]_i, [X]_e).
$$

$\mathrm{Na}^+/\mathrm{K}^+$-ATPase in neuron and astrocyte

$$
I_{NKA}^i = P_{NKA,i}\, \rho_{ox}\, f_{\mathrm{NKA}} \frac{[\mathrm{Na}^+]_i^{1.5}}{[\mathrm{Na}^+]_i^{1.5} + (\alpha_{NKA})^{1.5}} \frac{[\mathrm{K}^+]_e}{[\mathrm{K}^+]_e + (\beta_{NKA})},
$$

where

$$f_{\mathrm{NKA}} = 1.4\left(1 + 0.1245 \cdot \exp\left(-0.1\frac{FV_i}{RT}\right) + 0.0365 \cdot \sigma_{NKA} \cdot \exp\left(-\frac{FV_i}{RT}\right)\right),$$

$$\sigma_{NKA} = \frac{1}{7} \cdot \left(\exp\left(\frac{[\mathrm{Na}^+]_e}{67.3}\right) - 1\right),$$

$$\rho_{ox} = \frac{1}{1 + \exp\bigl((20 - [O_2]_e)/3\bigr)},$$

The corresponding currents are

$$I_{\mathrm{NKA}}^{\mathrm{Na}^+,i} = 3I_{\mathrm{NKA}}^i(t),$$
$$I_{\mathrm{NKA}}^{\mathrm{K}^+,i} = -2I_{\mathrm{NKA}}^i(t).$$

The K$^+$, Cl$^-$ cotransporter (KCC)

$$J_{\mathrm{KCl}} = \frac{\mathrm{RT}}{\mathrm{F}} \ln\left(\frac{[\mathrm{K}^+]_e[\mathrm{Cl}^-]_e}{[\mathrm{K}^+]_n[\mathrm{Cl}^-]_n}\right),$$

with corresponding currents

$$I_{\mathrm{KCl}}^{\mathrm{K}^+,n} = \mathrm{F}J_{\mathrm{KCl}}^n,$$
$$I_{\mathrm{KCl}}^{\mathrm{Cl}^-,n} = \mathrm{F}J_{\mathrm{KCl}}^n.$$

Astrocytic Kir4.1 channel

$$I_{Kir}^{\mathrm{K}^+,a} = P_{Kir} m_\infty \frac{[\mathrm{K}^+]_e}{[\mathrm{K}^+]_e + 13}(V_a - E_{\mathrm{K}^+}^a),$$

with

$$m_\infty = \left(2 + \exp\left(1.62\frac{\mathrm{F}}{\mathrm{RT}}(V_a - E_{\mathrm{K}^+}^a)\right)\right)^{-1}, \quad E_{\mathrm{K}^+}^a = \frac{\mathrm{RT}}{\mathrm{F}} \log\frac{[\mathrm{K}^+]_e}{[\mathrm{K}^+]_a}.$$

The Na$^+$, K$^+$, Cl$^-$ cotransporter (NKCC1)

$$J_{NKCC1}^a = P_{NKCC1}^a \frac{\mathrm{RT}}{\mathrm{F}} \log\left(\frac{[\mathrm{Na}^+]_e}{[\mathrm{Na}^+]_a}\frac{[\mathrm{K}^+]_e}{[\mathrm{K}^+]_a}\left(\frac{[\mathrm{Cl}^-]_e}{[\mathrm{Cl}^-]_a}\right)^2\right),$$

with corresponding currents

$$
\begin{aligned}
I_{\mathrm{NKCC1}}^{\mathrm{Na}^+,a} &= -\mathrm{F} J_{\mathrm{NKCC1}}^{a}, \\
I_{\mathrm{NKCC1}}^{\mathrm{K}^+,a} &= -\mathrm{F} J_{\mathrm{NKCC1}}^{a}, \\
I_{\mathrm{NKCC1}}^{\mathrm{Cl}^-,a} &= 2\mathrm{F} J_{\mathrm{NKCC1}}^{a}.
\end{aligned} \tag{8}
$$

The Na$^+$, Ca^{2+}-exchanger (NCX)

$$
I_{NCX}^{i} = P_{NCX}^{i} \left(\frac{[\mathrm{Na}^+]_e^3}{\alpha_{\mathrm{Na}^+}^3 + [\mathrm{Na}^+]_e^3} \right) \left(\frac{[\mathrm{Ca}^{2+}]_e}{\alpha_{\mathrm{Ca}^{2+}} + [\mathrm{Ca}^{2+}]_e} \right) \times \frac{\frac{[\mathrm{Na}^+]_i^3}{[\mathrm{Na}^+]_e^3} \exp\left(\frac{\eta \mathrm{F} V_i}{\mathrm{RT}}\right) - \frac{[\mathrm{Ca}^{2+}]_i}{[\mathrm{Ca}^{2+}]_e} \exp\left(\frac{(\eta-1)\mathrm{F} V_i}{\mathrm{RT}}\right)}{1 + k_{NCX} \exp\left(\frac{(\eta-1)\mathrm{F} V_i}{\mathrm{RT}}\right)}, \tag{9}
$$

with corresponding currents

$$
\begin{aligned}
I_{\mathrm{NCX}}^{\mathrm{Na}^+,i} &= 3 I_{NCX}^{i}, \\
I_{\mathrm{NCX}}^{\mathrm{Ca}^{2+},i} &= -I_{NCX}^{i}.
\end{aligned} \tag{10}
$$

Calcium-dependent parameters in the glutamate cycle

$$
\begin{aligned}
k_1 &= k_{1,\mathrm{max}} \frac{[\mathrm{Ca}^{2+}]_n}{[\mathrm{Ca}^{2+}]_n + K_{\mathrm{M}}}, \\
k_2(\mathrm{Ca}^{2+}) &= k_{20} + g(\mathrm{Ca}^{2+}) k_{2\mathrm{cat}}, \\
k_{-2}(\mathrm{Ca}^{2+}) &= k_{-20} + g(\mathrm{Ca}^{2+}) k_{-2\mathrm{cat}}, \\
g(\mathrm{Ca}^{2+}) &= \frac{[\mathrm{Ca}^{2+}]_n}{[\mathrm{Ca}^{2+}]_n + K_{\mathrm{Dv}}}.
\end{aligned}
$$

B Parameter fitting

The parameters needed for implementation of the GG-cycle cannot be sourced from literature due to a lack of data on timescales of glutamate and glutamine dynamics. Parameter selection is performed through parameter fitting, in which a loss function comprising two components is optimized. Firstly, we construct a loss function to ensure adequate timescales, i.e., the concentrations return to baseline after stimulation in adequate time. In order to so, we choose two time points in the simulation, where we consider ten data points at both time points. The first time point, t_1, is located right before the action potential. The second time point, t_2, is located after the action potential. The loss function is based on the differences between the values of the state

variables at both time points. To ensure that all variables are normalized, we multiply with the weight vector w_1, leading to the equilibrium loss defined as:

$$\text{Loss}_{\text{equi}} = \sum_{j=1}^{M=10} \sum_{i=1}^{N=27} \Big[w_{1,i} \left(X_i(t_{2,j}) - X_i(t_{1,j}) \right) \Big]^2$$

Secondly, we construct a loss function to ensure concentrations in a physiological range. To do so, we have constructed a range, denoted by r for each state variable, based on experimental data. In the loss function, we measure the distance of the variable to its physiological range. Subsequently, we multiply with a weight vector w_2 to normalize the variables, which can also be used to prioritize certain state variables. The loss function to ensure a physiological range is defined as:

$$\text{Loss}_{\text{phys}} = \sum_{i}^{N} \big[w_{2,i}\ d(X_i(t_1), r) \big]^2, \text{ where}$$

$$d(x, r) = \begin{cases} |\text{lower bound} - x| & \text{if } x < \text{lower bound} \\ 0 & \text{if } x \in \text{range } r \\ |x - \text{upper bound}| & \text{if } x > \text{upper bound} \end{cases}$$

All weights can be found in Table 1.

i	X_i	$w_{1,i}$	$w_{2,i}$	i	X_i	$w_{1,i}$	$w_{2,i}$	i	X_i	$w_{1,i}$	$w_{2,i}$
1	m	0	0	11	N_{N}	0	0	21	N^n_{Glu}	1000	100
2	h	0	0	12	N_{R}	0	0	22	$N^e_{O_2}$	0	0
3	n	0	0	13	N_{R_1}	0	0	23	N^n_{Gln}	1000	100
4	$N^n_{Na^+}$	0	0.001	14	N_{R_2}	0	0	24	N^a_{Gln}	1000	100
5	$N^n_{K^+}$	0	0.001	15	N_{R_3}	0	0	25	N^e_{Glu}	10^7	100
6	$N^n_{Cl^-}$	0	0.001	16	$N^a_{Na^+}$	0	0.001	26	m_{KDR}	0	0
7	W_n	0	0	17	$N^a_{K^+}$	0	0.001	27	h_{KDR}	0	0
8	$N^n_{Ca^{2+}}$	0	10^7	18	$N^a_{Cl^-}$	0	0.001	28	N^e_{Gln}	1000	100
9	N_{I}	0	0	19	W_a	0	0				
10	N_{D}	1000	1000	20	$N^a_{Ca^{2+}}$	0	0				

Table 1: Values of `weights1` and `weights2`

B.1 The optimization

In order to optimize the loss functions, the Borg Multiobjective Evolutionary Algorithm (BorgMOEA) algorithm from the global optimization Julia package BlackBoxOptim was used. The algorithm combines ε-dominance with Pareto solutions.

B.2 Sensitivity analysis

After optimizing the loss functions, we performed a sensitivity analysis to evaluate the robustness and reliability of the result. For the sensitivity analysis, we use the one-factor-at-a-time method (OAT). We increase and decrease each parameter by 5%, while keeping the other parameters fixed. Then we compute the loss function again to see if changing the parameter had a large influence on the result. We compute the sensitivity index as follows:

$$S_i = \left| \frac{L(p^*) - L(p)}{L(p)} \right|,$$

where $L(p)$ is the loss function for parameter p, and p^* is the parameter vector for 5% increase or decrease.

	φ_{GS}	φ_{SN}	φ_{SAT}	φ_{GM}	P^a_{EAAT}	$P^{l,a}_{Glu}$
$S_{i,min}$	0.3369	0.0033	0.0025	0.0910	0.2837	0.0034
$S_{i,max}$	0.2396	0.0034	0.0025	0.0080	0.2038	0.0039
$S_{i,total}$	0.5765	0.0067	0.0050	0.1790	0.4874	0.0072

Table 2: Sensitivity Indices with Min and Max Ranges

From the sensitivity indexes, we conclude that φ_{GS} affects the result the most, but all sensitivity indexes are sufficiently low. However, changing φ_{GS} results in a slightly different equilibrium but does not affect the timescale of the model, as can be seen in Figure 15. We conclude that the results obtained from parameter fitting are reliable and robust.

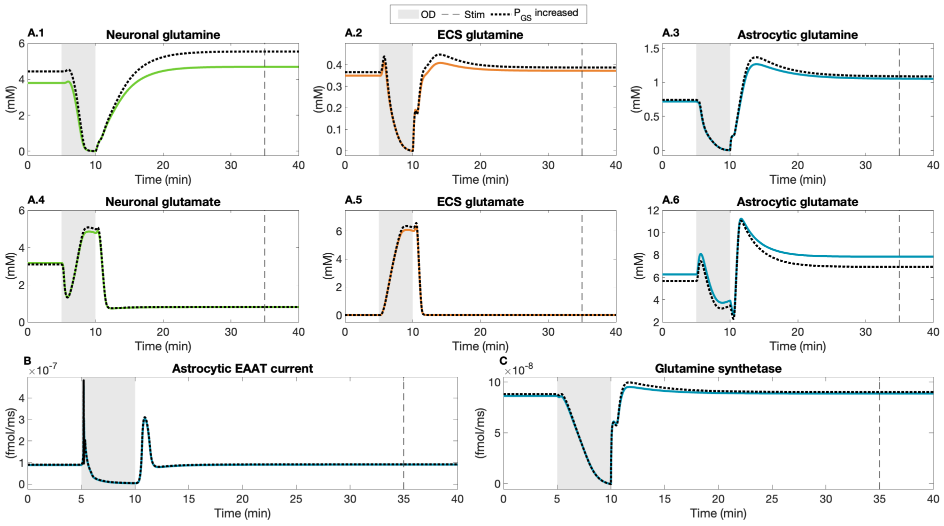

Figure 15: Comparison between default simulation of severe ischemia, and altered simulation with φ_{GS} increased by 5%.

C Additional figures

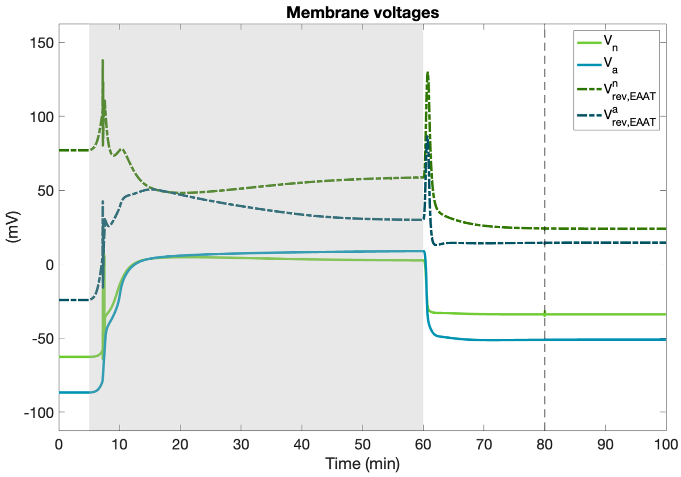

Figure 16: **Membrane potentials and EAAT reversal potentials during ischemia.**

D The complete model equations

D.1 Neuron

$$\begin{cases}
\frac{d}{dt}m &= \alpha_m(1-m)-\beta_m m,\\
\frac{d}{dt}h &= \alpha_h(1-h)-\beta_h h,\\
\frac{d}{dt}n &= \alpha_n(1-n)-\beta_n n,\\
\frac{d}{dt}N^n_{\mathrm{Na^+}} &= -\frac{1}{\mathrm{F}}\left(I_{\mathrm{G}}^{\mathrm{Na^+},n}+I_{\mathrm{L}}^{\mathrm{Na^+},n}+3I_{\mathrm{NKA}}^{\mathrm{Na^+},n}+3I_{\mathrm{EAAT}}^{\mathrm{Na^+},n}+3I_{\mathrm{NCX}}^{\mathrm{Na^+},n}\right)+W_nJ_{SAT}+\frac{1}{F}I_{\mathrm{stim}}(t),\\
\frac{d}{dt}N^n_{\mathrm{K^+}} &= -\frac{1}{\mathrm{F}}\left(I_{\mathrm{G}}^{\mathrm{K^+},n}+I_{\mathrm{L}}^{\mathrm{K^+},n}-2I_{\mathrm{NKA}}^{\mathrm{K^+},n}-I_{\mathrm{EAAT}}^{\mathrm{K^+},n}+I_{\mathrm{KCC}}^{\mathrm{K^+},n}\right),\\
\frac{d}{dt}N^n_{\mathrm{Cl^-}} &= \frac{1}{\mathrm{F}}\left(I_{\mathrm{G}}^{\mathrm{Cl^-},n}+I_{\mathrm{L}}^{\mathrm{Cl^-},n}-I_{\mathrm{KCC}}^{\mathrm{Cl^-},n}\right),\\
\frac{d}{dt}N^n_{\mathrm{Ca^{2+}}} &= -\frac{1}{2\mathrm{F}}\left(I_{\mathrm{G}}^{\mathrm{Ca^{2+}},n}-I_{\mathrm{NCX}}^{\mathrm{Ca^{2+}},n}+I_{\mathrm{L}}^{\mathrm{Ca^{2+}},n}\right),\\
\frac{d}{dt}W_n &= L_{H_2O}\mathrm{R\,T}\left([\mathrm{S}]_{\mathrm{n}}-[\mathrm{S}]_{\mathrm{e}}\right),
\end{cases}$$

D.2 Calcium-dependent glutamate cycle

$$\begin{cases}
\frac{d}{dt}N_{\mathrm{I}} &= -\frac{1}{\tau_{\mathrm{rec}}}N_{\mathrm{I}}+\frac{1}{\mathrm{F}}\left(I_L^{\mathrm{Glu},n}-I_{\mathrm{EAAT}}^{\mathrm{Glu},n}\right)+J_{\mathrm{GM}},\\
\frac{d}{dt}N_{\mathrm{D}} &= \frac{1}{\tau_{\mathrm{rec}}}N_{\mathrm{I}}-k_1N_{\mathrm{D}}+k_{-1}N_{\mathrm{N}},\\
\frac{d}{dt}N_{\mathrm{N}} &= k_1N_{\mathrm{D}}-(k_{-1}+k_2)N_{\mathrm{N}}+k_{-2}N_{\mathrm{R}},\\
\frac{d}{dt}N_{\mathrm{R}} &= k_2N_{\mathrm{N}}-(k_{-2}+3k_3[\mathrm{Ca^{2+}}]_n)N_{\mathrm{R}}+k_{-3}N_{\mathrm{R_1}},\\
\frac{d}{dt}N_{\mathrm{R_1}} &= 3k_3[\mathrm{Ca^{2+}}]_nN_{\mathrm{R}}-(k_{-3}+2k_3[\mathrm{Ca^{2+}}]_n)N_{\mathrm{R_1}}+2k_{-3}N_{\mathrm{R_2}},\\
\frac{d}{dt}N_{\mathrm{R_2}} &= 2k_3[\mathrm{Ca^{2+}}]_nN_{\mathrm{R_1}}-(2k_{-3}+k_3[\mathrm{Ca^{2+}}]_n)N_{\mathrm{R_2}}+3k_{-3}N_{\mathrm{R_3}},\\
\frac{d}{dt}N_{\mathrm{R_3}} &= k_3[\mathrm{Ca^{2+}}]_nN_{\mathrm{R_2}}-(3k_{-3}+k_4)N_{\mathrm{R_3}},
\end{cases}$$

D.3 Astrocyte

$$\begin{cases} \frac{d}{dt}m_{\mathrm{KDR}} & = \left(m_{\mathrm{KDR}}^{inf} - m_{KDR}\right) / \tau_{\mathrm{KDR}}, \\ \frac{d}{dt}h_{\mathrm{KDR}} & = \left(h_{\mathrm{KDR}}^{inf} - h_{KDR}\right) / \tau_{\mathrm{KDR}}, \\ \frac{d}{dt}N_{\mathrm{Na^+}}^{a} & = -\frac{1}{\mathrm{F}}\left(I_{T}^{\mathrm{Na^+},a} + 3I_{\mathrm{NKA}}^{\mathrm{Na^+},a} + I_{\mathrm{EAAT}}^{\mathrm{Na^+},a} + I_{\mathrm{NCX}}^{\mathrm{Na^+},a} + I_{\mathrm{L}}^{\mathrm{Na^+},a} - I_{\mathrm{NKCC1}}^{\mathrm{Na^+},a}\right) - A_a J_{\mathrm{SN}}, \\ \frac{d}{dt}N_{\mathrm{K^+}}^{a} & = -\frac{1}{\mathrm{F}}\left(-2I_{\mathrm{NKA}}^{\mathrm{K^+},a} + I_{\mathrm{EAAT}}^{\mathrm{K^+},a} + I_{\mathrm{Kir}}^{\mathrm{K^+},a} + I_{\mathrm{L}}^{\mathrm{K^+},a} - I_{\mathrm{NKCC1}}^{\mathrm{K^+},a} - I_{KDR}\right) +, \\ \frac{d}{dt}N_{\mathrm{Cl^-}}^{a} & = \frac{1}{\mathrm{F}}\left(I_{\mathrm{L}}^{\mathrm{Cl^-},a} + 2J_{\mathrm{NKCC1}}^{\mathrm{Cl^-},a}\right), \\ \frac{d}{dt}N_{\mathrm{Ca^{2+}}}^{a} & = -\frac{1}{2\mathrm{F}}\left(I_{\mathrm{L}}^{\mathrm{Ca^{2+}},a} - I_{\mathrm{NCX}}^{\mathrm{Ca^{2+}},a}\right), \\ \frac{d}{dt}W_a & = L_{H_2O}\mathrm{R\,T}\left([\mathrm{S}]_{\mathrm{n}} - [\mathrm{S}]_{\mathrm{e}}\right) \end{cases}$$

D.4 Glutamate-glutamine cycle, extracellular glutamate

$$\begin{cases} \frac{\mathrm{d}}{\mathrm{d}t}N_{\mathrm{Glu}}^{a} & = \frac{1}{\mathrm{F}}\left(I_{L}^{\mathrm{Glu},a} - I_{\mathrm{EAAT}}^{\mathrm{Glu},a} - I_{GS}\right), \\ \frac{\mathrm{d}}{\mathrm{d}t}N_{\mathrm{Gln}}^{a} & = -A_{PreSyn}J_{SN} + I_{GS}, \\ \frac{\mathrm{d}}{\mathrm{d}t}N_{\mathrm{Gln}}^{n} & = V_{PreSyn}\cdot J_{SAT} - J_{GM}, \\ \frac{\mathrm{d}}{\mathrm{d}t}N_{I} & = \frac{-N_I}{t_{rec}} + \frac{1}{F}(I_{Glu}^{l,n} - I_{EAAT}^{n}) + J_{GM}, \\ \frac{\mathrm{d}}{\mathrm{d}t}N_{\mathrm{Glu}}^{e} & = k_4 N_{\mathrm{R_3}} - \frac{1}{F}(I_{Glu}^{l,n} - I_{EAAT}^{n}) - \frac{1}{F}(I_{Glu}^{l,a} - I_{EAAT}^{a}) \end{cases}$$

D.5 Oxygen dynamics

$$\begin{cases} \frac{\mathrm{d}}{\mathrm{d}t}N_{O_2}^{e} & = -\frac{\alpha_{O_2}}{\mathrm{F}}\left(I_{\mathrm{NKA}}^{n} + I_{\mathrm{NKA}}^{a}\right) - J_{GS} + \frac{1}{2}\varepsilon_{O_2}W_e\log\Big(1 + \exp\big(2([O2]_{bath} - [O2]_e)\big)\Big). \end{cases} \tag{11}$$

E Model variables

Table 3: Model variables

Variable	Initial value	Unit	Description
Molar amounts			
$N^n_{Na^+}$	17.8	[fmol]	Neuronal sodium
$N^n_{K^+}$	288.1	[fmol]	Neuronal potassium
$N^n_{Cl^-}$	9.2	[fmol]	Neuronal chloride
$N^n_{Ca^{2+}}$	$8.5 \cdot 10^{-7}$	[fmol]	Neuronal calcium
N_{I}	$8.7 \cdot 10^{-7}$	[fmol]	Inactive glutamate state
N_{D}	$5.8 \cdot 10^{-3}$	[fmol]	Neuronal glutamate depot
N_{N}	$9.3 \cdot 10^{-4}$	[fmol]	Non-releasable glutamate pool
N_{R}	$1.9 \cdot 10^{-5}$	[fmol]	Ready-releasable glutamate pool
N_{R_1}	$3.6 \cdot 10^{-5}$	[fmol]	Vesicle bound-1 glutamate pool
N_{R_2}	$4.6 \cdot 10^{-5}$	[fmol]	Vesicle bound-2 glutamate pool
N_{R_3}	$1.2 \cdot 10^{-7}$	[fmol]	Vesicle bound-3 glutamate pool
$N^a_{Na^+}$	22.9	[fmol]	Astrocytic sodium
$N^a_{K^+}$	187.7	[fmol]	Astrocytic potassium
$N^a_{Cl^-}$	83.1	[fmol]	Astrocytic chloride
$N^a_{Ca^{2+}}$	$1.4 \cdot 10^{-7}$	[fmol]	Astrocytic calcium
$N^e_{O_2}$	31.2	[fmol]	Extracellular oxygen
N^n_{Gln}	0.0023	[fmol]	Neuronal glutamine
N^a_{Gln}	$5.1 \cdot 10^{-5}$	[fmol]	Astrocytic glutamine
N^e_{Glu}	$1.3 \cdot 10^{-8}$	[fmol]	Extracellular glutamate
N^e_{Gln}	$3.0 \cdot 10^{-4}$	[fmol]	Extracellular glutamine
Other variables			
m	0.024	-	Activation gating variable
h	0.97	-	Inactivation gating variable
n	0.0051	-	Activation gating variable
W_n	1.9	[1000μm^3]	Neuronal volume
W_a	1.8	[1000μm^3]	Astrocytic volume
m_{KDR}	0	-	Activation gating variable for KDR current
h_{KDR}	1	-	Inactivation gating variable for KDR current

Table 4: Currents, fluxes and transport rates

**Name**	**Unit**	**Description**
Neuron		
$I_{\mathrm{G}}^{X,n}$	pA	Voltage-gated current for ion $X = \{\mathrm{Na}^+, \mathrm{K}^+, \mathrm{Cl}^-, \mathrm{Ca}^{2+}\}$
$I_{\mathrm{KCC}}^{X,n}$	pA	$\mathrm{K}^+/\mathrm{Cl}^-$co-transporter current for $X = \{\mathrm{K}^+, \mathrm{Cl}^-\}$
I_{stim}	pA	External stimulation current
J_{SAT}	fmol/ms/mm^2	SAT transporter flux
J_{GM}	fmol/ms	Glutaminase transport rate
Astrocyte		
$I_{T}^{\mathrm{Na}^+,a}$	pA	Transient Na^+ channel current
$I_{\mathrm{Kir}}^{\mathrm{K}^+,a}$	pA	Kir4.1 K^+ channel current
I_{KDR}	pA	K^+ delayed rectifying current
J_{SN}	fmol/ms/mm^2	SN transporter flux
J_{GS}	fmol/ms	Glutamine synthetase transport rate
Both compartments, $i = \{n, a\}$		
$I_{\mathrm{L}}^{X,i}$	pA	Leak current for ion $X = \{\mathrm{Na}^+, \mathrm{K}^+, \mathrm{Cl}^-, \mathrm{Glu}, \mathrm{Ca}^{2+}\}$
$I_{\mathrm{NKA}}^{X,i}$	pA	$\mathrm{Na}^+/\mathrm{K}^+$-ATPase current for ion $X = \{\mathrm{Na}^+, \mathrm{K}^+\}$
$I_{\mathrm{EAAT}}^{X,i}$	pA	Excitatory amino acid transporter current for ion $X = \{\mathrm{Na}^+, \mathrm{K}^+, \mathrm{Glu}\}$
$I_{\mathrm{NCX}}^{X,i}$	pA	$\mathrm{Na}^+/\mathrm{Ca}^{2+}$-Exchanger current for $X = \{\mathrm{Na}^+, \mathrm{Ca}^{2+}\}$

F Model parameters

Table 5: Universal parameters
Dimensionless parameters are denoted with unit $'-'$.

Parameter	Value	Units	Description
F	96485.333	C/mol	Faraday's constant
R	8314.4598	(C·mV)/(mol·K)	Universal gas constant
T	310	K	Absolute temperature
z_{Na}	1	–	Valence of Na
z_{K}	1	–	Valence of K
z_{B}	1	–	Valence (unspecified)
z_{Ca}	2	–	Valence of Ca
z_{Glu}	-1	–	Valence of Glutamate
z_{Cl}	-1	–	Valence of Cl
z_{A}	-1	–	Valence (unspecified)
C	20	pF	Membrane capacitance
R_{H_e,H_a}	2/3	–	Proton ratio in neurons
$L_{\mathrm{H_2O},n}$	2×10^{-14}	$\mathrm{m^3/ms/bar}$	Neuronal water permeability
$L_{\mathrm{H_2O},a}$	2×10^{-14}	$\mathrm{m^3/ms/bar}$	Astrocytic water permeability
α_{O_2}	5.3/32	-	Conversion factor
ε_{O_2}	25×10^{-6}	$\mathrm{ms^{-1}}$	Oxygen diffusion rate
$\mathrm{Vol_{PreSyn,n}}$	1×10^{-3}	$\mathrm{mm^3}$	Presynaptic neuron terminal volume
$\mathrm{Vol_{PreSyn,a}}$	1×10^{-3}	$\mathrm{mm^3}$	Presynaptic astrocyte terminal volume
$\mathrm{Vol_{Cleft}}$	1×10^{-3}	$\mathrm{mm^3}$	Synaptic cleft volume
$\mathrm{r_{PreSyn,n}}$	$(\frac{3\cdot \mathrm{Vol_{PreSyn,n}}}{4\pi})^{1/3}$	mm	Radius of presynaptic neuronal terminal
$\mathrm{r_{PreSyn,a}}$	$(\frac{3\cdot \mathrm{Vol_{PreSyn,a}}}{4\pi})^{1/3}$	mm	Radius of presynaptic astrocytic terminal
$\mathrm{A_{PreSyn,n}}$	$4\pi \cdot \mathrm{r^2_{PreSyn,n}}$	$\mathrm{mm^2}$	Area of presynaptic neuronal terminal
$\mathrm{A_{PreSyn,a}}$	$4\pi \cdot \mathrm{r^2_{PreSyn,a}}$	$\mathrm{mm^2}$	Area of presynaptic astrocytic terminal

Table 6: Ion Permeabilities and Conductances

Parameter	Value	Units	Description
**Neuronal Channels**			
$P^{t,n}_{\mathrm{Na}^+}$	80×10^{-4}	$\mathrm{mm}^3/\mathrm{ms}$	Transient Na^+ channel permeability
$P^{l,n}_{\mathrm{Na}^+}$	2×10^{-6}	$\mathrm{mm}^3/\mathrm{ms}$	Leak Na^+ permeability
$P_{d,\mathrm{K},n}$	400×10^{-6}	$\mathrm{mm}^3/\mathrm{ms}$	Delayed rectifier K^+ permeability
$P^{l,n}_{K^+}$	20×2^{-5}	$\mathrm{mm}^3/\mathrm{ms}$	Leak K^+ permeability
$P^{g,n}_{Cl^-}$	19.5×10^{-6}	$\mathrm{mm}^3/\mathrm{ms}$	Gated Cl^- permeability
$P^{l,n}_{Cl^-}$	2.5×10^{-6}	$\mathrm{mm}^3/\mathrm{ms}$	Leak Cl^- permeability
$P^{g,n}_{Ca^{2+}}$	1.6×10^{-5}	$\mathrm{mm}^3/\mathrm{ms}$	Gated Ca^{2+} permeability
$P^{l,n}_{Ca^{2+}}$	4×10^{-10}	$\mathrm{mm}^3/\mathrm{ms}$	Leak Ca^{2+} permeability
$P^{l,n}_{Glu}$	1×10^{-7}	$\mathrm{mm}^3/\mathrm{ms}$	Leak Glu permeability
**Astrocytic Channels**			
$P^{l,a}_{Ca^{2+}}$	4×10^{-11}	$\mathrm{mm}^3/\mathrm{ms}$	Leak Ca^{2+} permeability
$P^{l,a}_{Glu}$	1×10^{-10}	$\mathrm{mm}^3/\mathrm{ms}$	Leak Glu permeability
$P^{l,a}_{Na^+}$	1×10^{-7}	$\mathrm{mm}^3/\mathrm{ms}$	Leak Na^+ permeability
$P^{l,a}_{K^+}$	70×10^{-6}	$\mathrm{mm}^3/\mathrm{ms}$	Leak K^+ permeability
$P^{l,a}_{Cl^-}$	1.5×10^{-6}	$\mathrm{mm}^3/\mathrm{ms}$	Leak Cl^- permeability
g_{KDR}	3566.4	$\mathrm{pS}\ /\mu\mathrm{m}^2$	KDR conductance
P_{KDR}	40	mm^2	KDR scaling

Table 7: Transporter and Recycling Parameters

Parameter	Value	Units	Description
**NKA Pump**			
α_{NKA}	13.0	mM	Intracellular Na^+ half-sat. conc.
β_{NKA}	0.2	mM	Extracellular K^+ half-sat. conc.
$P_{\text{NKA},n}$	110	pA	NKA pump strength (baseline)
$P_{\text{NKA},a}$	90	pA	NKA pump strength (astrocyte)
**Glutamate Recycling**			
$k_{1,\text{max}}$	1.4×10^{-4}	ms^{-1}	Max forward reaction rate
K_M	0.0023	mM	Ca^{2+} half-sat. conc.
K_{DV}	0.1	mM	Forward reaction saturation
k_{20}	2.1×10^{-5}	ms^{-1}	Uncatalyzed forward rate
$k_{2,\text{cat}}$	0.02	ms^{-1}	Catalyzed forward rate
$k_{\text{min},20}$	1.7×10^{-5}	ms^{-1}	Uncatalyzed backward rate
$k_{\text{min},1}$	5×10^{-5}	ms^{-1}	Backward reaction rate
k_3	4.4	mM ms^{-1}	Forward reaction rate
$k_{\text{min},3}$	0.056	ms^{-1}	Backward reaction rate
k_4	1.45	ms^{-1}	Vesicle fusion rate
t_{rec}	50	ms(fmol)^{-1}	Vesicle fusion factor
**EAAT Transporters**			
$P_{\text{EAAT},n}$	42/9	–	Maximal neuronal EAAT strength
$\alpha_{\text{EAAT},n}$	0.0032	A/m^2	Neuronal EAAT fitting parameter.
$\beta_{\text{EAAT},n}$	0.0288	mV^{-1}	Neuronal EAAT fitting parameter.
$P_{\text{EAAT},a}$	42	–	Maximal astrocytic EAAT strength
$\alpha_{\text{EAAT},a}$	0.0032	A/m^2	Astrocytic EAAT fitting parameter.
$\beta_{\text{EAAT},a}$	0.0288	mV^{-1}	Astrocytic EAAT fitting parameter.
rg	5×10^{-4}	mM^{-1}	Glutamate release fitting param.
sg	9×10^{-2}	mM	Glutamate release fitting param.
**NCX Transporter**			
$P_{\text{NCX},n}$	35	pA	Maximal NCX current
$P_{\text{NCX},a}$	1	pA	Astrocytic NCX current
$\alpha_{\text{NCX,Na}}$	87.5	mM	Na^+ half-saturation conc.
$\alpha_{\text{NCX,Ca}}$	1.38	mM	Ca^{2+} half-saturation conc.
e_{NCX}	0.35	–	Energy barrier position
$k_{\text{sat,NCX}}$	0.1	–	NCX saturation factor
**Other transporters**			
P_{Kir}	0.1	nS	Inward-rectifier K^+ conductance
$K_{\text{Ce,thres}}$	13	mM	Extracellular K^+ threshold concentration
$P_{\text{NKCC1},a}$	33×10^{-7}	fmol/(ms·mV)	Astrocytic NKCC1 transporter strength
U_{KCC}	1.3×10^{-6}	fmol/(ms·mV)	KCl cotransporter strength

Table 8: Parameters for Glutamate-Glutamine Cycle and Transporters

**Parameter**	**Value**	**Units**	**Description**
φ_{GS}	3×10^{-7}	fmol/ms	Glutamine synthetase flow rate
φ_{SN}	6×10^{-5}	fmol/1000μm^2·ms	SN transporter flux constant
φ_{SAT}	1.7×10^{-4}	fmol/mm^3/ms	SAT transporter flux constant
φ_{GM}	1×10^{-7}	fmol/ms	Glutaminase flow rate
$K_{\mathrm{m,GM}}^{\mathrm{Gln}}$	0.6	mM	Michaelis-Menten constant for glutamine
$K_{\mathrm{m,GS}}^{\mathrm{Glu}}$	2.5	mM	Michaelis-Menten constant for glutamate
$K_{\mathrm{m,GS}}^{\mathrm{NH_4}}$	0.2	mM	Michaelis-Menten constant for NH_4^+
$K_{\mathrm{m,GS}}^{\mathrm{ATP}}$	2.3	mM	Michaelis-Menten constant for ATP
$K_{\mathrm{m,SN}}^{\mathrm{Gln}}$	1.57	mM	Michaelis-Menten constant for glutamine
$K_{\mathrm{m,SN}}^{\mathrm{Na}}$	31	mM	Michaelis-Menten constant for Na^+
$K_{\mathrm{m,SN}}^{\mathrm{H}}$	100×10^{-6}	mM	Michaelis-Menten constant for H^+
$K_{\mathrm{m,SAT}}^{\mathrm{Gln}}$	0.3	mM	Michaelis-Menten constant for glutamine
$K_{\mathrm{m,SAT}}^{\mathrm{Na}}$	10	mM	Michaelis-Menten constant for Na^+
$\mathrm{NH}_{4,a}^+$	0.15	mM	Astrocytic ammonium concentration
H_a	6.3096×10^{-5}	mM	Astrocytic proton concentration
H_e	6.3069×10^{-5}	mM	Extracellular proton concentration
